# Aquaculture Production of the Brown Seaweeds *Laminaria digitata* and *Macrocystis pyrifera*: Applications in Food and Pharmaceuticals

**DOI:** 10.3390/molecules26051306

**Published:** 2021-02-28

**Authors:** Diane Purcell-Meyerink, Michael A. Packer, Thomas T. Wheeler, Maria Hayes

**Affiliations:** 1 Cawthron Institute, 98 Halifax Street, Nelson 7010, New Zealand; Mike.Packer@cawthron.org.nz (M.A.P.); Tom.Wheeler@cawthron.org.nz (T.T.W.); 2 Food BioSciences, Teagasc, Ashtown, Dublin 15, Ireland; Maria.Hayes@teagasc.ie

**Keywords:** aquaculture, seaweed, *Laminaria digitata*, *Macrocystis pyrifera*, extraction, food, pharmaceuticals, feed

## Abstract

Seaweeds have a long history of use as food, as flavouring agents, and find use in traditional folk medicine. Seaweed products range from food, feed, and dietary supplements to pharmaceuticals, and from bioenergy intermediates to materials. At present, 98% of the seaweed required by the seaweed industry is provided by five genera and only ten species. The two brown kelp seaweeds *Laminaria digitata*, a native Irish species, and *Macrocystis pyrifera*, a native New Zealand species, are not included in these eleven species, although they have been used as dietary supplements and as animal and fish feed. The properties associated with the polysaccharides and proteins from these two species have resulted in increased interest in them, enabling their use as functional foods. Improvements and optimisations in aquaculture methods and bioproduct extractions are essential to realise the commercial potential of these seaweeds. Recent advances in optimising these processes are outlined in this review, as well as potential future applications of *L. digitata* and, to a greater extent, *M. pyrifera* which, to date, has been predominately only wild-harvested. These include bio-refinery processing to produce ingredients for nutricosmetics, functional foods, cosmeceuticals, and bioplastics. Areas that currently limit the commercial potential of these two species are highlighted.

## 1. Introduction

### 1.1. Laminaria digitata and Macrocystis pyrifera in the Context of Global Seaweed Aquaculture

Nearly three hundred seaweed species of interest have been identified for their potential commercial value [1], yet only ten are cultivated extensively with a handful of other species grown for niche applications. These include three brown seaweeds *Saccharina japonica*, *Undaria pinnatifida*, and *Sargassum fusiforme* (Ochrophyta, Phaeophyceae); four red seaweeds *Neopyropia*/*Pyropia/Porphyra* spp., *Eucheuma* spp., *Kappaphycus alvarezii*, and *Gracilaria* spp. (Rhodophyta); and five green seaweeds *Ulva clathrata* (formerly *Enteromorpha clathrata*), *Monostroma nitidum* and *Caulerpa* spp., *Ulva* spp., *Oedogonium termedium* (Chlorophyta) [2]. The brown seaweed commonly called Japanese kelp, *Saccharina japonica*, formerly known as *Laminaria japonica*, was the most cultivated seaweed in the world until 2010. It still retains a considerable market share, commanding 29% of global production in 2014 and over 33% in 2018 [3,4]. However, in 2010, production of *Eucheuma/Kappaphycus* surpassed 9.07 million tonnes with a value of over EUR 1,079 million [5], and by 2014, *Eucheuma* (35%) and *Kappaphycus* (6%), collectively at 41% global production, were the most cultivated species [3]. In the context of the cultivation advantage gained by aquaculture, seaweed cultivation is unequalled in mariculture, as 94% of the annual seaweed biomass used globally is from cultivated sources [6]. 

At present 98% of seaweed cultivated across the globe comes from five genera: *Saccharina, Undaria, Neopyropia/Pyropia/Porphyra, Eucheuma/Kappaphycus*, and *Gracilaria* [4,5,7,8]. These species are predominantly cultivated at sea, with a few groups including kelps and nori requiring an extra step, often onshore, to facilitate their microscopic life cycle stage. This step, known as the aquaculture hatchery phase, enables growth and seeding of ropes prior to deployment at sea [9]. 

Seaweed products range from food to pharmaceuticals, and bioenergy intermediates to materials. Brown and green seaweeds are predominately used in food as a source of fibre, protein, and minerals, especially throughout Asia [2]. Red seaweeds have been used as food, and as a source of agars and carrageenan which are used in food, cosmetic ingredients, and for biomedical applications [10]. The global seaweed industry is worth more than USD 6 billion per annum, of which 85% is for human consumption, and seaweed-based polysaccharides (carrageenan, agar, and alginates) account for nearly 40% of the world’s hydrocolloid market [2]. In Europe, brown seaweeds were traditionally used to produce additives (e.g., alginates) or animal feeds in the form of meal [11]. *L. digitata* and *M. pyrifera* are two brown seaweed species that are harvested and cultivated globally. In comparison to other kelp species such as *Saccharina japonica, Saccharina latissima*, and *Undaria pinnatifida, M. pyrifera* is a native species to New Zealand whereas *Undaria pinnatifida* is an invasive species and *Saccharina japonica* is not a native species [12,13]. *L. digitata* is a native Irish species with a wide distribution at low water on the Irish coast, when compared to *Saccharina latissima*, also a native Irish species, yet not comparable to *L. digitata* in distribution and abundance on the Irish coast [14,15]. In 2016, *L. digitata* global wild harvest yielded ~45,000 tonnes, and *M. pyrifera* yield reached 31,835 tonnes; however, only one tonne of *M. pyrifera* was produced through aquaculture [3]. Chile was the highest producer of brown seaweed from natural populations at 300,000 dry tonnes per year by 2012 [16], in a global context 7% of the brown seaweed from natural populations was provided by *Macrocystis* sourced in Chile and Mexico [17]). Seaweed is sourced mainly from wild harvesting, with only 2.4% from cultures, which are dominated by *Agarophyton chilense* [18]. It can be observed from these data that *M. pyrifera* when compared with *L. digitata* has significant future cultivation potential through aquaculture. Additionally, this aquaculture potential may assist in a reduction in the impact of wild harvesting on *M. pyrifera*’s distribution off the Chilean coast.

Specifically, *L. digitata* has been used in Europe as a food supply for algivores in the mariculture of abalone and sea urchins; it has also been harvested and supplied to Asia as a dried product and used as stock for soup making [14]. Within the fuel and renewable energy sector, it has been investigated for methane gas production through bioconversion trials in France and the US [14]. In Australia, Chile, and the US, *M. pyrifera* was also used to feed abalone [14,19,20]. In Mexico, *M. pyrifera* was used as a meal for goats. The digestibility of this seaweed was 77% when fed to this ruminant animal [21]. Digestibility increased to 85% when fed to male bovine zebu bulls [22]. Due to its low digestibility in salmonids, *M. pyrifera* in a derived flour form was added as a food supplement at 1.5%, 3%, and 6% dry weight (DW) of the total diet as a mineral and carbohydrate source [23]. 

In the US, commercial harvesting of *L. digitata* started in 2010, among several other species, and now kelp aquaculture is considered one of the fastest growing industries in the North Eastern US [24]. A native species of the North Atlantic coast *L. digitata* commonly called *oarweed* was used in an offshore system cultivation trial in Ireland between 2008 and 2011. The trial found that its production using this system was commercially viable [15]. In Europe, *L. digitata* has been the main raw material used to supply the French alginate industry, being harvested mainly from the upper sublittoral zone and around the coast of Brittany and surrounding islands. In Norway, *L. digitata* grows in large masses at the lower end of the eulittoral zone and had been previously an important industry in Norway until seaweed market expansion required increased biomass and was replaced by *Laminaria hyperborea (L. hyperborea*) forests [25]. In Iceland, *L. digitata* was grown to supply the UK alginate industry which is based in Scotland [25]. In 2009, alginate production was moved from Scotland to Norway by Pronova, a Norwegian alginate producer, but *L. hyperborea* is still sourced in Scotland and Ireland and *Ascophyllum nodosum* is also sourced in Scotland, Ireland, Iceland, and Norway for Pronova [26]. Recently, the UK’s interest in alternatives to fossil fuels saw the inclusion of *L. digitata* in method development for bioethanol production [27]. 

In 2012, Asia dominated the cultivation of *Laminaria* species. The three main producers were China, Korea, and Japan, with China’s market share at 4.35 million tonnes, or 23% of total global production. At this time Denmark was the only European country to cultivate *Laminaria* [28]. Global consumption of the *Laminaria* genus as a food or a feed additive accounted for over 0.9 million tonnes of the biomass produced in 2012, worth between USD6 billion and 8 billion, or 50% of the total seaweed global revenue for 2012 [1].

*Macrocystis pyrifera*, commonly called the giant kelp, is presently wild harvested in Alaska. However, aquaculture started in 1972 in California where an offshore kelp farm was set up. It achieved limited success due to design problems, low nutrient supply, and storms yet persevered until 1982 [24]. Work continued in California on *M. pyrifera* between 1980 and 1986, when another group received funding and managed not only to cultivate it effectively by modelling their system on a natural kelp bed, but also created a planting technology using fertiliser in oligotrophic waters, and performed genetic studies creating 800 strains [29]. The US Marine Biomass Program discontinued funding of projects after 12 years and USD 20 million, as seaweed which had been grown specifically as an alternative fuel source to fossil fuels proved to be uneconomical by 1986 [30]. In Southern Argentina, *M. pyrifera* grows to depths of 55 m; collections of *M. pyrifera* were made from these large kelp beds, primarily for alginate production, until this practise ceased in the early 2000s [25,31]. *Macrocystis pyrifera* (formerly *M. angustifolia*) was cultivated in South Africa on an experimental scale, as a potential resource for alginate production and abalone feed [25]. Northern Chile has also harvested *M. pyrifera* initially in smaller quantities than California, yet exploitation of these natural kelp beds has increased in Chile in recent years to supply food to the abalone industry. This demand has initiated pilot aquaculture cultivation research of *M. pyrifera* to a potential productivity level of 200 tonnes (fresh)/ha/year [32]. A further study including economic profitability calculations noted that a cultivation system had to be of 30–50 ha in size and *M. pyrifera* priced at EUR 64/tonne was essential to reach economic viability [19]. More recent work has found that a 10-ha cultivation system with *M. pyrifera* priced at EUR 72/wet tonne would be profitable [33] *Macrocystis pyrifera* was only cultured in Peru, with Chile the predominant country for wild harvest collection followed by the US. Peru has one of the most productive marine coastlines in the world, with over 34 recognised brown seaweeds found there; 4% of annual seaweed biomass landings in Peru are produced through seaweed farms, yet this has been declining since 2012 [34]. The US has used *M. pyrifera* harvested off the Californian coast as a source of alginate since at least 1913; the harvests have varied from 325,157 wet tonnes in 1918, to 214 tonnes in 1931, to 90,718 in 1984 [35]. In comparison with *Laminaria*, Chile’s *Macrocystis* harvest was 23,587 tonnes [28]. *Macrocystis pyrifera*, similar to *Laminaria*, are used as foods or food additives [1].

This review describes the state of play of brown seaweeds in the context of global seaweed production and outlines the present seaweed studies in the literature on the two brown kelp species *M. pyrifera* and *L. digitata*. In addition to previous reviews, it examines the differences that occur when one species, *M. pyrifera*, is predominantly wild harvested, versus *L. digitata* which has been grown extensively via aquaculture, and the impact that has on the variety of products being sourced and developed from each species. It details the best practice to be applied to aquaculture cultivation of these two species, and lists the food, feed, and pharmaceutical products produced from *M. pyrifera* and *L. digitata.* Additionally, it makes suggestions where potential improvements could be applied to expand the seaweed bioproducts sector.

### 1.2. Aquaculture methods for Brown Kelps

#### 1.2.1. Macrocystis pyrifera

The aim of seaweed aquaculture is to meet the global demand for seaweed which cannot be fulfilled from wild harvest of seaweed alone. World production of farmed seaweeds doubled between 2000 and 2012; the FAO reported a global increase of over 20 million tonnes of seaweed production since 2001 [10,28]. This growth has coincided with the largest increase in global aquaculture, where almost 10 million tonnes of seaweed product came from aquaculture between 2011 and 2015 [10]. In 2015, of the total 30.4 million tonnes of seaweed produced, the aquaculture industry produced 29.4 million tonnes with only 1.1 million tonnes produced from wild harvest [10]. The predominant cultivators of seaweed are countries throughout Asia and a market which has shown considerable expansion is Indonesia [28]. 

Other countries have been investigating and trialling the most efficient cultivation systems to produce commercially efficient seaweed cultivation. A proposed Canadian offshore kelp farm producing *Laminaria* sp. in close proximity to a salmon farm was studied using a mathematical model [36]. This proposed 1060 m of ropes, on either end of a salmon cage. The annual biomass yield from the model was 1600 kg dry weight, and it was deemed that a return on investment would be obtained after 6 years of seaweed introduction on the farm. Additionally, if increased kelp production occurred on several salmon farms, this would result in improved productivity of kelp and a profitable net return. Environmental impacts such as nitrogen and oxygen levels did not increase above background levels. Alternative species were also suggested for cultivation including *Macrocystis* and *Nereocystis* [36]. Another study noted that *M. pyrifera* protein content increased from 9 to 13% when cultured in proximity to salmon farms in Chile [37]. This finding is potentially economically advantageous as *M. pyrifera* is used as a food source for abalone [14,19,20].

Extensive work on cultivation of *M. pyrifera* in Chile has been essential to protect the natural kelp beds still present which have been harvested extensively. Best practice for efficient production of *M. pyrifera* requires starting with healthy juvenile sporophytes. This has been optimised to a growing period of only 45 days, a reduction from 60 days. Wild-sourced sori are used as seed material and once sporulation has occurred and a spore density of 40,000 cells ml^−1^ has been reached, sterile spores are transferred to 10 L rectangular tanks containing polyvinyl chloride (PVC) cylinders wrapped in 1.5 mm nylon string. Once settlement has occurred after 24 h, they are transferred to new clean 10 L tanks with seawater enriched with Provasoli’s Enriched Seawater (PES) culture media. These cultures are maintained at an optimum photoperiod of 16:8 light day cycle, temperature of 12 °C, photo irradiance of 12 µmol^−1^ s^−1^ m^2^, and aeration rate of 414 L h^−1^. After 45 days, juvenile sporophytes of 4–5 mm in size are produced and can be harvested for human consumption at this stage. If being grown to produce biomass for fuel, these optimised conditions enable the open ocean growing season to be extended by a month, increasing biomass [38]. 

The optimal out-planting method uses long lines of 50–100 m in length. These juvenile sporophytes are visible as a brown “fuzz” on the nylon lines at this stage and can be transferred to long lines by wrapping the nylon tightly around the long lines, which are a rope-based material. The long lines are then suspended in the nearshore environment, secured at both ends with floatation buoys to maintain buoyancy in the water column and then weights maintain position [39]. This out-planting method is similar to the traditional method used in East Asia. 

A second method of nearshore cultivation uses individual juvenile kelps, produced from a free-floating cultivation of the sexual phase (gametophytes) producing unattached, floating sporophytes, which are grown in tanks for several months, to a size of about 8 cm. These are then attached manually to long lines [40]. Comparisons between these two cultivation methods in locations in Chile have found this second method produced up to three times the biomass than the previous method [41]. The authors conclude that the increased productivity is due to these gametophyte plants being in a unialgal environment, resulting in less impact of bacteria and disease as they grow. Additionally, the increased size to 8 cm in comparison to 4–5 mm (juvenile sporophytes) means they can outgrow the epifauna and epiflora that they encounter on introduction to the nearshore environment [41]. 

#### 1.2.2. Laminaria digitata 

*Laminaria digitata* is a common kelp predominantly found along the North European and Eastern American coast [14]. Similar to *M. pyrifera*, it has a complex life cycle that includes a microscopic gametophyte phase of growth requiring a hatchery system to support efficient reproduction for farming of the macroscopic growth phase. The sporophytes are then out-planted in the near-shore ocean environment [15]. This cultivation method is specific to *L. digitata* practiced off the Irish coast. 

This Irish study comprehensively outlines the best practice to follow when cultivating *L. digitata* throughout its life cycle. This study, “The seaweed hatchery project”, was carried out in Ireland over a two-year period. It investigated the potential of *L. digitata* as a commercial seaweed aquaculture crop using new techniques and improving existing practices. Conditions for establishing optimum growing gametophytes requires wild-sourced sori tissue collected on a low spring tide in the lower intertidal and subtidal regions off the Irish coast. This sori tissue is then cleaned, all epiphytes are removed, and then it is placed in the cold (~−10 °C) dark for 18–24 h. Then, these pieces are chopped further into smaller pieces of 4–5 cm in size and are placed in a 1 L beaker with sterile seawater for 35–40 min to allow zoospore release, being stirred occasionally with a sterile glass rod. When the water appears cloudy, this indicates spores have been released. The rehydrated sori tissue is then filtered out and transferred to zoospore solution in a suitable culture vessel. The solution is then aerated in PES media which are changed every two weeks. Light is provided, the plantlets are covered in red cellophane, and irradiance at the surface of the glassware is approximately 15–20 µmol m^−2^ s^−1^, with day length starting from a 16:8 light–dark cycle then progressing to 24 h of complete light and a temperature of 10 °C. Cultures are maintained for 3–5 months to produce optimal biomass for spraying of the macroscopic growth phase of juvenile sporophytes onto the string [15].

Prior to out-planting, reproduction of the gametophytes must be induced. This is achieved by supplying new media, changing the light spectrum from red to blue, maintaining the incubation conditions described in the previous paragraph but reducing the day length to a 12:12 light–dark cycle. These conditions are maintained for 12–15 days or until reproductive structures, i.e., fertilised eggs or developing sporophytes, are observed. At this stage, the culture is ready to be sprayed onto string. The string, in 30 cm lengths, is wrapped around 65 mm of square plastic PVC drainpipe that has 4–5 cm holes made in it in as many areas as possible without reducing the structural integrity of the collector. Once the culture is sprayed, it must immediately be placed vertically into tanks in the hatchery for 30–45 days. Conditions are maintained at 10 °C, day length at 12:12, but irradiance and aeration are incrementally increased. Irradiance starts at 35–40 µmol m^−2^ s^−1^ from day 0 to 3, then increases to 60–70 µmol m^−2^ s^−1^ for the remaining 30–45 d. No aeration occurs from day 0 to 3, then from day 3 to 14, it is turned on to a low setting and is progressively increased to a moderate setting. After day 14, aeration is increased towards a high setting. Deployment of the now grown juvenile sporophytes at sea is the same method as that used from *M. pyrifera*, with the nylon being wrapped around large anchored and floated longlines between October and December. These lines are maintained and checked every two months with optimum harvest after 5–6 months, usually in April–May (Northern hemisphere spring), just before the water temperature increases, to avoid seaweed being infested with epiphytes [15].

Optimum growing depth was 2 m for out-planting unialgal gametophyte cultures of *L. digitata* in Helgoland, North Sea, Germany, with optimum blade growth noted in spring and summer. *L. digitata* also had a longer growing season, only reducing growth in September compared to July for both *L. hyperborea* and *S. latissima* (formerly *L. saccharina*), potentially indicating an adaptation to life in the sublittoral fringe [42].

Wave exposure impact on growth rates of *L. digitata* found a reduction in blade growth at the lowest and highest wave velocities, which may occur as a trade-off to increase blade strength [43]. A comparable wave velocity study on *Undaria pinnatifida* sporophytes on seeding strings noted that total biomass increase was significantly higher than total length, indicating seawater velocity encourages greater biomass productivity over length increase in sporophytes [44]. Alginate is a component of the cell wall of brown seaweeds and is partly responsible for the flexibility of the seaweed; therefore, species which grow in more exposed and turbulent sites usually contain higher alginate content [25]. *L. digitata* is found in the upper sublittoral zone in rocky wave-exposed locations, and it is considered to have a high alginate content [14,25].

Several *L. digitata* studies have looked specifically at the impact of temperature and produced guidelines regarding tolerance levels for this species. For the microscopic gametophyte growth phase, its temperature range is quite restricted with reductions in growth at 20–21 °C, with death observed after one week at 22–24 °C [42,45]. Other studies from Helgoland, North Sea, Germany found that optimal temperatures for vegetative gametophyte growth were 10–18 °C, during a 16:8 light–dark cycle to simulate summer photoperiods, with induction of gametogenesis at between 5 and 15 °C, and maximum sporophyte development during summer photoperiods with enriched nutrient regimes, even though the fastest gametogenesis was at 10–15 °C and the highest sporophyte recruitment was at 5 °C [46]. Previous work found sporophytes were tolerant to conditions in North European waters, with optimum growth from 5 to 15 °C, but at 20–22 °C, a 25% reduction in growth rate occurs [47]. Projected increase in sea temperatures due to climate change will significantly impact the *L. digitata* populations on the European coast, with model projections predicting a push to the verge of local extinction in the first half of the 21st century, consequently causing a decline in species whose life cycle depends on this seaweed for survival [48].

## 2. Current and Future Uses 

### 2.1. Food and Feed Uses of Brown Seaweeds 

Seaweeds are traditionally cooked and used as sea vegetables in Asian countries, yet their consumption by Western countries is minimal [49]. The nutritional content of fats, proteins, and carbohydrates in brown seaweeds depends on the season and location of harvest. Specifically, fat content in seaweeds is less than 5% dry weight (DW), with *L. digitata* and *S. latissima* harvested in Ireland containing only 1% and 0.5% fat, respectively [14,50]. Protein percentage values range from 5 to 20%. *L. digitata* collected off the UK coast had a protein content of 15.9%(DW) [51], and *Undaria pinnatifida*, commonly called wakame, collected in spring on the northwest Iberian coast, had a protein content of 16.8% of seaweed DW [52]. Carbohydrates account for 13–60% of the DW of brown seaweeds [53,54]. A study of four brown species sampled off the Isle of Seil in Scotland, on eight occasions between August 2010 and October 2011, found carbohydrate content ranged from 34.6 ± 3.1%, 33.2 ± 3.8%, 28.5 ± 3.9, and 37.4 ± 4.0% DW in *L. digitata, L. hyperborea, Saccharina latissima*, and *Alaria esculenta*, respectively. Interestingly when protein content was compared between the species, the values were higher than previously reported in the literature at 6.9 ± 1.1% in *L. digitata*, 6.8 ± 1.3% in *L. hyperborea*, 7.1 ± 1.7% in *Saccharina latissima*, and 11.0 ± 1.4% in *Alaria esculenta*. Results noted the range of protein levels were highest in the first quarter and lowest in the third quarter of the year [55], indicating potential seasonal impact on protein levels detected in this study. 

The contribution of brown seaweeds to the food and animal feed industry has been primarily as whole foods or as additives to feeds. In 2009, the 78,109 tonnes of hydrocolloids that were traded included 58% carrageenan and 11% agar, both from red seaweed and 31% alginate from brown seaweed [56]. The main commercial seaweed extracts are hydrocolloids which include agar, alginates, and carrageenans. The main use of alginates is as thickening or gelling agents and emulsion stabilisers [56]. Hydrocolloids sourced from algae are called phycocolloids. The most useful phycocolloid derived from brown seaweed in the food industry is alginate [25]. The phycocolloids found in the brown seaweeds *Macrocystis sp.*, *Laminaria sp.*, and *Ascophyllum sp.* include the alginates which are a soluble source of fibre [57]. *L. digitata* total fibre content when compared to whole foods provides 6.2 g/100 g wet weight; this value exceeds other whole foods including brown rice (3.8 g/100 g weight) with the exception of brown lentils (8.2 g/100 g weight) [58,59]. In comparison to the dietary fibre guideline daily allowance (GDA) set by the American Association of Analytical Chemists (AOAC) fibre recommendation, using an 8 g portion size, *L. digitata* can provide 12.5% of that fibre [58]. Fucoidan is another phycocolloid and soluble fibre source predominantly found in the brown seaweed species *Saccharina religiosa* (formerly *Laminaria religiosa*) and *Nemacystus decipiens* [57]. Fucoidan is an acidic heteropolysaccharide, and the biological activities of fucoidan are multi-factorial. Its biological activity is utilised through its low molecular weight and sulphate groups. Structural skeletal characterisation is still needed to locate specific branching sites of the sulphate groups [60]. 

*M. pyrifera* and *L. digitata* were used previously to produce alginic acid powder for use in diet biscuits to provide a feeling of fullness [25]. Both species were utilised for alginate production of gels for the food industry [25,61]. Alginate extracted from *M. pyrifera* has also been used as a stabiliser for food products such as ice cream, yogurt, and cream, as well as in foods as an emulsifier and gelling agent for sauces and dressings [62]. A food study used *M. pyrifera*, also called “huiro” in Chile, as an ingredient in Chilean recipes, specifically for huiro fritters and breadstick recipes to test whether seaweeds improved nutritional content, compared with the usual ingredients. Between 3 and 28% of the ingredients were replaced using dried ocean-sourced *M. pyrifera*; the results found that protein was lower in the huiro fritters at 6.9% DW, but the breadsticks with huiro showed high protein levels at 9.5% of DW. This study did taste test all products yet products were not considered a commercial success [63]. *Saccharina japonica*’s dried seaweed powder extract was added at 1% to breakfast sausage ingredients and an improvement in physiochemical and sensory properties of the sausages was noted as well as improving the ash content [64]. Table 1 lists the current commercial feed, food, and functional food products produced from *M. pyrifera* and *Laminaria* spp. 

Seaweeds or sea vegetables are a great source of B-group vitamins (mainly B_1_, B_12_), along with the lipophilic vitamin A (derived from the carotenoid β-carotene) and vitamin E (tocopherol). *M. pyrifera* contains quantities of α-tocopherol (the most biologically active form of vitamin E) comparable and in some cases higher than plant oils which are considered to be abundant in this vitamin, such as sunflower seed and soybean oil [65,66]. *M. pyrifera* and *L. digitata* were used to produce crude alginate for use as binding agents for salmon feeds [25]. *M. pyrifera* provided a carbohydrate and mineral supplement in a flour-derived meal ingredient added as 3% DW of the salmonid fish species diet [23]. Juvenile white shrimp were given a *M. pyrifera*-based diet supplement. The dose was calculated based on shrimp body weight at a concentration of (33.3 g/kg) of *M. pyrifera*, then 1.6 g of this concentration was fed to the shrimp over 28 days; a protein efficiency level of 1.7 was recorded [67]. *M. pyrifera* is also used to feed abalone as an in situ ocean-based food comprising 9–13% crude protein [14,19,20]. *L. digitata* was also used as a fresh ocean-based food source for the algivores, abalone and sea urchin, in Ireland (Table 1) [14]. *M. pyrifera* was used in ruminant diets due to its high digestibility rates. When incorporated at 30% DW of the goat diet, it had a 70% digestibility value; for bovine zebu bulls, a meal supplement provided 8.5% of their dietary protein, and this achieved 85% digestibility [21,22]. Two *Laminaria* species, *L. digitata* and *L. hyperborea*, provided a complete daily diet of 1.4 kg wet weight (WW) for six North Ronaldsay sheep in Scotland resulting in 79.6% digestibility [68]. *L. digitata* powder was also used as a dietary supplement (at a concentration of 0.001 kg per day) for rabbits [69] (Table 1).

The main uses of *L. digitata* have been for laminarin and fucoidan extract generation [14]. In France, it is predominately used to supply the alginate industry. The alginate content of brown seaweeds from Irish waters is 32% DW and fucoidan accounts for up to 15% DW, yet this is dependent on the extraction method used [14,57]. In addition to alginates, *Laminaria* species have additional polysaccharides with commercial value; in comparison to *M. pyrifera, L. digitata* contains 32.2% alginic acid, 5.5% fucoidan, 14.4% laminarin, and 13.3% mannitol [57].

Earlier studies on weaning pigs and piglets used a combination of laminarin, fucoidan, and ash to supplement diets for improved growth performance. Laminarin and fucoidan were tested with a range of lactose concentrations within the daily diet of weaning pigs for 21 days to see the effect on weight, average daily gain (ADG), and food conversion ratio (FCR). Results noted that the inclusion of the laminarin and fucoidan extract may reduce the need for high lactose diets of animals less than 60 kg in weight, and lessen other common problems which occur in pigs post weaning [70] (Table 1). 

More recently, seaweed extracts have been commercially patented by companies such as Ocean Harvest Technology based in Ireland and Olmix based in France, to name just two. Ocean Harvest Technology have made a seaweed extract from brown, green, and red seaweeds to produce OceanFeed Swine^®^ (OFS), which was tested in Chile on 1809 pigs. OFS was supplied as a flour supplement at 5 g/kg dose per day from day 21 to 55 of swine age. Results indicated their daily average weight gain (ADG) increased by 26 g and improved their feed efficiency (FE) by 0.07. In addition, there was an increase in the slaughter weight to (92.38 kg ± 0.47) from the control group weight of (90.97 ± 0.47 kg), showing statistical significance with a *P*-value of (<0.05). A reduction in the bacteria *Escherichia coli* and an increase in *Lactobacillus* sp. were also observed in these pigs [71]. Olmix have made a piglet feed supplement called Ecopiglet using the green seaweed *Ulva* sp. They tested 833 piglets between day 5 and day 21 of life with a 50 g dose of Ecopiglet per animal twice a day. Results noted no change in the ADG, survivability, or microbial gut community but did noticed a significant decrease in observed diarrhoea incidence [72].

Other studies on pigs have supplemented sow diets with a 0.001 kg/day seaweed extract consisting of laminarin (0.001 kg), fucoidan (0.0008 kg), and ash (0.0082 kg). This seaweed extract was administered to ten sows for 107 days of gestation, followed by 24 days of neonatal piglet growth. The study found piglets had enhanced immune function and colonic microflora at weaning [73]. Laminarin and fucoidan extract from *L. digitata* was added to the basal diet of weaning pigs in a 1.5 g/kg dietary supplement. Similar to the sow study, the bacterial microflora of the colon improved with a reduction in enterobacteria, bifidobacteria and lactobacilli populations in the caecum and colon, yet unlike the sow study, only marginal effects on the immune response were noted [74]. β-glucans are complex polysaccharides extracted from *L. digitata.* They were added as a pig dietary supplement at 250 mg/kg of body weight, which lead to a reduction in the Enterobacteriaceae population and pro-inflammatory markers in the pig’s colon [75]. An *L. digitata*-based extract with 500 mg/kg of laminarin and 420 mg/kg of fucoidan was used to supplement pig diets for 21 days pre-slaughter. A 75% reduction in lipid oxidation in muscle tissue, which improved pork steak quality, was observed [76] (Table 1).

### 2.2. Pharmaceutical Uses of Brown Seaweeds 

Pharmaceuticals are well-defined molecules that are used for medical purposes to cure, treat, or prevent disease [78]. The use of marine algae is noted in the Chinese “Materia Medica” of Shen-nung 2700 B.C. Seaweeds were used in folk medicines for the treatment of goitre, nephritic diseases, anthelmintic, catarrh, vermifuge, and skin diseases [79]. The use of brown seaweeds to treat medical ailments were documented in the 1750s by a physician who used ash from kelp fronds, which is rich in iodine, to treat goitre [80].

Phycocolloids are used in wound dressings as a gelling agent to assist in blood coagulation; *M. pyrifera* and *L. digitata* provide sodium and calcium alginates with a range of viscosities from low to high which enable wound dressings to stop bleeding by assisting blood clotting [25,81]. Alginic acid powder has been used for relieving acid indigestion and to treat gastroesophageal reflux (GERD) disease [25,61]; calcium alginate beads were used to control the release of medicinal drugs and other chemicals and are sourced from *M. pyrifera* and *L. digitata* [25,82]. Sodium alginate from *L. digitata* is used to produce a soft and elastic gel, which may be used in microparticles to aid drug delivery [83] (Table 2). The current commercial pharmaceutical products from *M. pyrifera* and *Laminaria* spp. are listed in Table 2.

Fucoidans are fucose-rich sulphated polysaccharides found in the extracellular matrix (ECM) of brown seaweeds. Purported fucoidan properties include anti-inflammatory, antioxidant, antibacterial, lipid inhibition, and immunological activities [84,85,86,87]. *L. digitata* was one of the brown species where extracts from it exhibited strong antithrombotic, anti-inflammatory activities, and a decrease in tumour proliferation, with results of an 80% (*P*-value, 0.01) reduction in tumour cell adhesion to human platelets under static conditions [85]. Another laminaria species, *Saccharina japonica*, was found to cause a significant reduction in thrombus lysis time when oral administration of fucoidan (molecular weight 300 kDa) at a dosage of 400 mg per day for 5 weeks was carried out [88]. Fucoidan extracted from *M. pyrifera* has also been reported to be a powerful immune modulator, enabling delays in apoptosis and promotion of pro-inflammatory cytokine production in human neutrophils at low concentration (2 × 10^5^), as well as activation of dendritic cells (DCs) and T-cells [89] (Table 2).

Fucoidan is also used to alleviate metabolic syndrome, and benefits angiogenesis and bone health [60]. Metabolic syndrome (MetS) commonly refers to the pathological state in which proteins, fats, carbohydrates, and other substances in the body are metabolically disordered. These disorders are the pathological basis of cardiovascular and cerebrovascular disease and diabetes. To treat MetS, a multi-drug treatment approach is required yet individual risk factors remain unmanageable [90]. Natural products including marine polysaccharides have been claimed to reduce MetS. Fucoidan can alleviate MetS-related disorders, including obesity, hyperlipidaemia, hyperglycaemia, and hypertension. Fucoidan has been intensively investigated as a potential hypoglycaemic agent to assist in type 2 diabetes treatment; specifically, fucoidan extracted from the brown seaweed *Fucus vesiculosus* can be used in the treatment of type 2 diabetes [91]. Low molecular weight fucoidan (LMWF) has been reported to possess bioactive compounds which can protect vascular endothelial function and reduce the basal blood pressure in diabetic rats. Therefore, fucoidan is a possible candidate drug for protection of the endothelium in diabetic cardiovascular complications [92]. In protection of the gastrointestinal tract, fucoidan’s pharmacological activity was discovered in the treatment of inflammatory bowel disease (IBD). Fucoidan was able to reduce crypt destruction and mucosal damage in the colon of dextran sodium sulphate-treated mice to treat chronic colitis [93]. Fucoidan from seaweed *Cladosiphon okamuranus* improved chronic colitis by downregulating the expression of the proinflammatory cytokine IL-6 in the colonic epithelial cells of IBD mice [94].

Fucoidan inhibits angiogenesis through control of the expression of vascular endothelial growth factor (VEGF) and endothelial cell plasminogen activator inhibitor-1. The brown seaweed *Sargassum fusiforme* produces a fucoidan that can inhibit the angiogenesis of human microvascular endothelial cells in a dose-reliant manner [95]. Regarding the improvement of bone health, fucoidan is thought to reduce blood vessel development in bone tumours such as osteosarcomas [96]. Low molecular weight fucoidan (LMWF), extracted from fresh *Sargassum hemiphyllum*, increased bone density and ash weight in C57 BL/6J female mice. These findings allow conclusions to be drawn regarding fucoidan as a promising osteogenic drug [97].

A crude polysaccharide-rich seaweed extract from *L. digitata* was tested for its effect on metabolic activity of human gut microbiota. Results observed improved gut microbiota composition and increased short-chain fatty acids [98]. An *M. pyrifera* extract-derived product range was proposed on a US patent in 2012; the products included a high purity fucoidan 75–90% kelp oil and/or kelp concentrate, and one product had 50% krill oil added. These products were proposed to provide total antioxidant protection as pharmaceutical products [99]. Several *L. digitata* pharmaceutical products, also based on seaweed extract, which act as moisturising agents for the skin with protecting, soothing, and smoothing properties and another product, which can be used as a homeopathic remedy, are produced by two companies: Actipone^®®^ and Boiron [100,101] (Table 2).

Secondary metabolites are primarily excretory products made under different stress situations, such as exposure to ultraviolet (UV) radiation, variations in temperature and salinity, or environmental contaminants. The main secondary metabolites manufactured in algae tissues are phenolic compounds, halogenated compounds, sterols, terpenes, and small peptides, as well as other bioactive compounds [53,102,103,104]. At present, more than one hundred metabolites have been identified within forty-nine species of brown seaweed [105]. 

Phlorotannins are a large and varied group of naturally occurring polyphenolic compounds and are also secondary metabolites found in brown seaweeds [106]. Phlorotannins are tannin derivatives made of several phloroglucinol units connected to each other in distinct ways [103,107]. Phlorotannins, display effective antioxidant activity through their ability to scavenge reactive oxygen species [108]. Furthermore, due to the inhibitory effect of hyaluronidase (HAase) activation, phlorotannins exhibit antiallergic, bleaching, anti-wrinkle, and skin antiaging actions [105]. Phlorotannins are components of cell walls and are also found in vesicles in the cytoplasm (physodes). These compounds can make up to 20% of the dry weight of seaweeds [109]. *Fucus* spp., *Sargassum* spp., and *Ascophyllum nodosum* are three brown seaweeds with the highest phenolic compounds which range from 12.2 to 14% DW; when compared, *L. digitata*’s range was considerably lower, between ~0.2 and 5.3% DW of phenolic compounds [53,106]. *Fucus spiralis* was found to have higher molecular weight phlorotannins, which usually exhibit the strongest lipid peroxidation inhibitory activity, when compared to three other brown seaweeds: *Gongolaria nodicaulis* (formerly *Cystoseira nodicaulis*), *Ericaria selaginoides* (formerly *Cystoseira tamariscifolia*), and *Gongolaria usneoides* (formerly *Cystoseira usneoides* [110]. Recent work on *L. digitata* found that its phlorotannin content was ~4.5% of dry matter [111]. Phlorotannins from *Saccharina japonica* were found to be effective in proliferation control of human tumour cells [112]. *Laminaria hyperborea* phlorotannins exhibited efficacy at wound sealing and reconstruction during wound healing. Additionally, the application of photosynthetically active radiation (PAR), and PAR + UV radiation, induces a high-stress response and resulted in an increase in physodes in the epidermal cells of the seaweed frond, and a resulting photoprotective response [113]. *M. pyrifera* has also been found to contain two phlorotannins, phloroeckol and tetrameric phloroglucinol, both demonstrating antidiabetic and antioxidant activity as well as preventing skin aging [114] (Table 2).

### 2.3. Other Uses of Brown Seaweeds

*M. pyrifera* and several *Laminaria* spp. have been used for other purposes beyond the food, feed, and pharmaceutical industries. The renewable energy and fuel industry have investigated the ability to use the polysaccharides laminarian and mannitol to make ethanol from *L. hyperborea* [117]. Both *M. pyrifera* and *Saccharina latissima* also known as *Laminaria saccharina* Linnaeus Lamour, have been investigated for methane production mostly via anaerobic fermentation. The mannitol and alginate content of the kelp was key to methane production; the higher the mannitol content, the better gas yield produced [25]. *Saccharina latissima* was also converted to methane using anaerobic fermentation, with methane yield doubling from autumn to spring. This study found laminarian and mannitol levels were reduced to 5% yet alginate was only reduced to 30% by the anaerobic fermentation methane yield and was dependent on the total carbohydrate content of the raw seaweed [118] (Table 3). Other current uses of *Macrocystis* sp. and *Laminaria* sp. are listed in Table 3.

Removal of toxic metal ions has been successfully achieved using both *Macrocystis* sp. and *Laminaria* sp. as dried seaweed to trap metal ions in solution [119,120]. Additionally, *L. digitata* contains calcium alginate that was shown to effectively remove copper and cadmium from single and binary solutions [121] (Table 3).

In the cosmetics industry, alginates from *M. pyrifera* and *Laminaria* sp. have been used for their gelling, emulsion stabilising properties, and more recently, their immunostimulating properties [82,122]. Specifically, laminarian from *Laminaria* sp. is used in cosmetics for its ability to act as an antioxidant, anticellulite, and anti-inflammatory agent [104]. Nutricosmetics are defined as products and/or ingredients which are nutritional supplements for the care of the skin, nails, and hair. They function by working within the body to promote beauty from within [123]. A high purity fucoidan rich extract from *M. pyrifera* was proposed in a patent in 2012 by a company called KNOCEAN Sciences, Inc. It is claimed to be an antioxidant booster and health and wellness additive to be added to cosmetics, functional foods, and pharmaceuticals [99] (Table 3). 

### 2.4. Functional Foods Applications

#### 2.4.1. Protein Content and Applications

A nutritional study in Chile catalogued the amino acid content of three edible Chilean seaweeds: *Codium fragile* (Chlorophyta), *Agarophyton chilense* (formerly *Gracilaria chilensis*) (Rhodophyta), and *M. pyrifera*, which contained proteins at 13.7–10.8% and amino acid contents at 1879.6–1417.7 mg/100 g dry algae. Specifically, *M. pyrifera* had 13.2 ± 0.30%DW protein [66]. Protein sourced from seaweed contains all essential amino acids, and specifically glycine, alanine, arginine, proline, glutamic, and aspartic acids. In seaweeds, their essential amino acids account for almost half of the total amino acids and their protein profile is similar to that of egg protein [124,125]. *Laminaria* sp. contains 13 g of aspartic acid/16 g N and 24 g glutamic acid/16 g N, and when compared to other brown seaweed species *Undaria pinnatifida* and *Sargassum fusiforme* (formerly *Hizikia fusiforme*), *Laminaria* sp. had significantly higher aspartic acid content [125]. Seaweed as an alternative protein source to animal protein has been postulated [126]. In the context of functional foods, the addition of seaweed supplements to improve the nutritional value of a food to deliver essential amino acids appears to have potential. Regarding regulations, in the US, the Food and Drug Administration (FDA) uses the designation GRAS (generally recognised as safe), and both *M. pyrifera* and *L. digitata* are listed as GRAS for human consumption as flavour enhancers and flavour adjuvants, with concentrations in food not exceeding current good manufacturing practice (GMP) [127]. Lectins and phycobiliproteins are two protein families which have notable bioactive properties and have been detected in seaweeds [128,129]; however, brown seaweeds do not contain either lectins or phycobiliproteins. 

#### 2.4.2. Carbohydrate Content and Applications

The carbohydrate content of *M. pyrifera* was reported as 75.3 ± 0.2% DW previously [66]. When compared to the daily consumption of fruit and vegetables, this resembles the carbohydrate contribution of dried fruit [130]. 

#### 2.4.3. Tocols Applications

Tocols are liposoluble metabolites which include α-, β-, γ-, and δ-tocopherol and their isomers α-, β-, γ-, and δ-tocotrienols. They are made by plant cells with antioxidant action, and in the human body, α-tocopherol acts as vitamin E [66]. Alpha tocopherol was tested at a dose of (50 mg/day) as a dietary supplement and showed potential to prevent prostate and colorectum cancer. A 34% reduction in prostate cancer and 16% reduction in colorectum cancer was observed, yet was not effective at treating stomach cancer where a 25% increase in cancer occurred [131]. Recent work comparing three edible seaweeds in Chile found the total tocols content for *Codium fragile, Agarophyton chilense*, and *M. pyrifera* ranged between 391.9 and 1617.6 µg/g lipids, with the highest value for *Codium fragile*, the lowest for *Agarophyton chilense*, and *M. pyrifera* reaching 1457 µg/g lipids. The most abundant fatty acid found in *M. pyrifera* was α-tocopherol at 1327.7 ± 4.4 µg/g lipid [66]. The lipid fraction of *M. pyrifera* can be considered to have a high tocol content when compared to arachis oil, grapeseed oil, palm oil, and sunflower seed oil. Interestingly, *M. pyrifera* contains 1327.7 ± 4.4 µg/g of α-tocopherol, compared to seed oils such as arachis oil which contains between 49 and 373 µg/g α-tocopherol, grapeseed oil 16–38 µg/g α-tocopherol, palm oil 4–193 µg/g α-tocopherol, soyabean 9–352 µg/g α-tocopherol, and sunflower seed oil 403–935 µg/g α-tocopherol, which is the closest α-tocopherol content to *M. pyrifera* [65,66]. *M. pyrifera* had only 0.7 ± 0.3% DW lipid content, yet the value of vitamin E is important, as it provides stability to the PUFA present in this seaweed, preventing the development of free radicals and therefore, converting this seaweed into a potential complimentary food in light of its important contribution of vitamin E and PUFA [66].

#### 2.4.4. Pigment Applications

Pigments including β-carotene have been recommended as dietary supplements to prevent cardiovascular disease (CVD) and cancer. One study recommended ≥0.4μmol/l β-carotene and ≥0.5μmol/l α+β-carotene for primary prevention of CVD and cancer [132]. Conversely, another study on β-carotene tested the effectiveness of supplementation at 20 mg/d on cancer prevention in 29,133 male smokers between the ages of 50 and 69 for 5–8 years. Results found an increase in lung (18%), prostate (23%), and stomach (25%) cancers. This study concluded that β-carotene supplements at high doses should be avoided by smokers, who also had high alcohol consumption, as it may increase the incidence of lung cancer, yet additional advice for smokers was the most effective mode of action which was to stop smoking to avoid lung cancer [131,133]. The potential causal mechanism between β-carotene supplements and alcohol consumption to increase lung cancer risk have observed ethanol-related changes in carotenoid metabolism, and hepatocellular toxicity in response to β-carotene supplements when consumed with extremely high alcohol intake (i.e., 50% of calories as ethanol), yet further work is needed as to how these responses would enhance the risk of lung cancer [134,135]. These studies highlight the use of β-carotene as a dietary supplement primarily for prevention of disease; the contributary factors such as smoking 20 cigarettes a day and drinking 11 g of alcohol a day increase the incidence of developing lung disease, potentially outweighing the preventative impact of a dietary supplement such as β-carotene [131,133].

The principal carotenoid in *M. pyrifera* is β-carotene. When compared to other edible seaweed species in the same study, β-carotene was lowest in *M. pyrifera* at 17.4 mg/g dry algae, highest in *Codium fragile* at 197.9 mg/g dry weight, with *Agarophyton chilense* in the middle at 113.7 mg/g beta carotene in dry algae [66]. The α-tocopherol and β-carotenoid contents in *M. pyrifera* are beneficial because they can perform as both vitamins and antioxidants [66]. The literature recommends a 15 mg/day dose of β-carotene to contribute to a healthy diet, and so 75 g of *Codium fragile* would complement the daily recommended requirements of β-carotene [136]. Regarding optimal times to harvest seaweed species, β-carotene and tocopherol isolated from *Saccharina japonica* were highest from July through to September and less during the winter [137].

#### 2.4.5. Phlorotannins Applications

*M. pyrifera* contains the secondary metabolites, phlorotannins, and specifically phloroeckol and tetrameric phloroglucinol, which display both antidiabetic effect and antioxidant activity and can contribute to the prevention of skin aging [114]. Phloroeckol has also been reported to prevent Alzheimer’s disease, yet this has only been tested using phlorotannins from the brown seaweed *Ishige okamurae* [138]. 

#### 2.4.6. Polysaccharides—Soluble Fibre Phycocolloids 

Dietary fibres are complex carbohydrates. They are mainly sourced in vegetables, fruits, grains, nuts, and root crops and are a vital part of a healthy diet. Since dietary fibre is not digested by digestive enzymes, it does not provide direct nutrition in the human body. Yet, dietary fibre indirectly helps human nutrition by involving it in some important functions to promote digestive health during its passage through the gastrointestinal track. These functions consist of reduction in incidences of colorectal cancers, suppression of bowel inflammations and related abdominal disorders, facilitation of bowel movement, and growth promotion of health-promoting gut microflora. Recommended average daily intake of dietary fibre is between 25 and 30 g in the US and >18 g in the UK [139]. The health benefits of seaweed-derived dietary fibre have been focussed mainly on components in humans, and specifically on potential anti-obesity effects, such as enhanced repletion, delayed nutrient absorption, and delayed gastric emptying, yet the effects of whole seaweeds containing alginate appear to be restricted [140,141,142]. In the US, dietary fibre is considered a nutrient under the Nutrition and Education Act of 1990 [143].

Fucoidans are L-fucose-containing sulphated polysaccharides found in the cell walls of brown algae. Structures of fucoidans vary as does their complexity amongst different species. Generally, brown seaweed fucoidans consist of one of two types of homofucose backbone chains, either the repeating α(1->3)-linked l-fucopyranose residues or an alternation of α(1->3) and α(1->4)-linked l-fucopyranosyls, which, in either case, may be substituted with sulphate or acetate and/or have side branches containing fucopyranoses or other glycosyl units, e.g., glucuronic acid [85,144]. The first type of fucoidan backbone was isolated from seaweeds *Saccharina latissima, Chorda filum, Cladosiphon okamuranus*, and *L. digitata* [145]. Other fucoidans reported in the literature include small amounts of various other monosaccharides, e.g., glucose, galactose, xylose, and/or mannose [144]. 

At present, fucoidan’s profile as a diet supplement from disease assistance is improving due to the extensive preclinical testing being undertaken. Its typical activities include antitumour, antioxidant, anticoagulant, anti-inflammatory, antiviral, and immunoregulatory [60]. Fucoidan is also used to alleviate metabolic syndrome, for protection of the gastrointestinal tract, and for benefiting angiogenesis and bone health [60]. In protection of the gastrointestinal tract, fucoidan’s pharmacological activity was discovered in the treatment of inflammatory bowel disease (IBD). Fucoidan was able to reduce crypt destruction and mucosal damage in the colon of dextran sodium sulphate-treated mice to treat chronic colitis [93]. Fucoidan from seaweed *Cladosiphon okamuranus* improved chronic colitis by downregulating the expression of the proinflammatory cytokine IL-6 in the colonic epithelial cells of IBD mice [94].

Laminarin is another polysaccharide which contains soluble fibre in the form of phycocolloids; brown seaweeds noted for their laminarin content are *Saccharina japonica* and *Saccharina latissima* [57]. When comparing the fibre content of foods from terrestrial plants, seaweed has similar or even higher levels of dietary fibre. The average total dietary fibre content in seaweed can vary from 36 to 60% based on its dry matter [146]. Almost 55–70% of its total dietary fibre is represented by the soluble fibre fraction which primarily contains agar, alginates, and carrageenan at varying amounts depending on the type of seaweed and the seasonal growing conditions [6,53,147,148,149]. The brown seaweed genera, *Fucus* and *Laminaria*, have the highest content of insoluble dietary fibre among the other commercially harvested seaweed used in the food industry [150]. The typical daily portion size of the seaweeds consumed in Asian cuisines on a dry matter basis is about 8 g [57]. Seaweed can provide 12–15% of daily dietary requirements of fibre in the human diet, with brown seaweeds contributing the highest amount at 14.28%, reds providing the lowest at 10.64%, and greens slightly higher at 12.10% [148]. This is a large amount compared to that of other food sources when compared on a weight-for-weight basis [57]. 

#### 2.4.7. Polysaccharides—Prebiotic Potential

Brown seaweeds have been investigated for their bioactive properties. A bioactive compound is a substance that has a biological activity [151] linked to its ability to regulate one or more metabolic processes, which results in the promotion of improved health conditions [152].

*L. digitata* is rich in polysaccharides that function well as prebiotics to improve human gut biota populations [98]. Prebiotics are compounds in food that induce or support the growth or activity of microorganisms such as bacteria and fungi deemed beneficial to a host [153]. This is most often by consumption of the prebiotic by the microorganism as carbon sources. Laminarins sourced from *L. digitata* have been used in several studies to access their effectiveness as prebiotics. One study on rats used a treatment of 1 g of laminarins from *L. digitata.* The results noted no changes to gut biota; however, an increase occurred in the colon luminal mucin content, and also a decrease in luminal mucin in the jejunum, ileum, and the caecum [154]. Another study which used laminarin isolated from *L. digitata* found significant changes in gut biota with an increase in parabacteroides, fibrobacter, and lachnospiracease and a decrease in streptococcus, ruminococcus, and peptostreptococcaceae [98]. *M. pyrifera* does not contain Laminarin, which is only found in the brown seaweed genera *Laminaria* and *Fucus* [155].

Fucoidans from brown seaweed have shown bioactivity including, e.g., anti-inflammatory, antioxidant, antibacterial, and immunological activity; lipid inhibition; obesity prevention or treatment [84,85,86]. Unlike laminarins, fucoidans from brown seaweeds have not shown any prebiotic characteristics useful for improving gut biota communities.

#### 2.4.8. Polyunsaturated Fatty Acids 

The carbon-18 (C_18_) polyunsaturated fatty acids (PUFA) have been found in seaweeds and plants and are important in human and fish nutrition as neither can synthesize them. They are exceptional sources of n-3 fatty acids with 18 to 20 or more carbons, such as eicosapentaenoic acid (EPA) and docosahexaenoic acid (DHA) [156,157]. Yet, fish can lengthen and desaturate dietary fatty acids (18:2n-6 and 18:3n-3) [158]. The health benefits of n-3 fatty acids, also known as omega-3 fatty acids EPA and DHA, are numerous and include proper foetal development and neuronal, retinal, and immune function [159,160]. Other potential uses of EPA and DHA are prevention of mild Alzheimer’s disease and obesity [159,161,162,163,164,165]. Seasonal impacts on the PUFA content in *Saccharina japonica* have found the PUFA, (n-6) family, was highest during warm months, while (n-3) PUFAs were highly abundant during the colder months when seaweed thalli were very young; a decrease occurs progressively toward October when sori development was noted (Hafting, 2015) [137]. Fatty acid methyl esters (FAMES) analysis on *M. pyrifera* found that the highest PUFA present was linoleic acid (18:2n-6) at 43.41%; it also detected the monounsaturated fatty acids (MUFA) and found 18:1n-9c (oleic acid) was highest at 19.64% [66]. The advantage of this seaweed is that it contains a suitable PUFA n-6/n-3 relation of high impact in human and animal nutrition [66].

## 3. Current Extraction Strategies

### 3.1. Seaweed Cell Wall Structure

**Figure 1:**
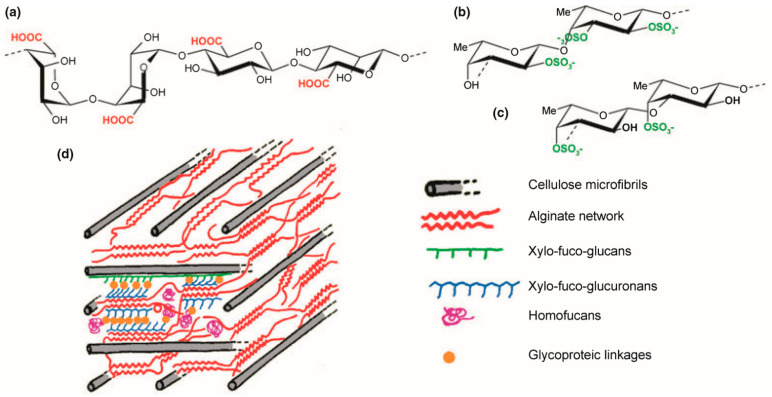
The main polysaccharide structures of brown alga: (**a**) alginate; (**b**) sulphated fucan from Fucales; (**c**) sulphated fucan from Ectocarpales; (**d**) proposed model of the biochemical organisation of brown alga cell wall structure. Reproduced with permission from [166].

The cell wall structure of seaweeds requires considered approaches to break and remove the complex polysaccharides without losing the vital proteins compounds of interest. Protein content in brown seaweeds varies from 5 to 20% dry weight [53,54,167]. Brown algae have evolved a cell wall (Figure 1) which shares elements with both plants and animals due to its multicellular eukaryotic nature. The evolution of an extracellular matrix (ECM) enabled development, established defence systems conferring innate immunity, and provided a boundary for nonself recognition [168]. In eukaryotes such as seaweed, this ECM was organised usually as a three-dimensional network of fibres embedded in fluid components. The cell wall structure of brown seaweeds contains plant cellulose, yet these crystalline fibres only account for 1–8% of the thallus (DW) [169]. The cell wall main components are anionic polysaccharides, i.e., alginates and fucoidans [168,170,171,172]. Alginates consist of two uronic acids, β-1,4-d-mannuronate and α-1,4-l-guluronate, arranged in blocks along the polysaccharide chain (Figure 1a) [166].

### 3.2. Functional Food Extraction

#### 3.2.1. Protein Extraction

*M. pyrifera* was studied for its nutritional value in Chile with two other species—*Codium fragile* and *Agarophyton chilense*. The study analysed *M. pyrifera*’s protein content using a proximal analysis standard method for proteins (N 6.25, AOAC 954.01) [173]. Additionally, the amino acids were analysed using a simple and fast HPLC method [174]. The sample was dried and milled prior to extraction. The samples were then ground further using a mortar and pestle. A sample equivalent to 2 mg of protein was weighed into a hydrolysis tube, then 4 ml of 6.0 M hydrochloric acid. The internal standard used was d,l-α-aminobutyric acid. The solution was gassed with nitrogen and sealed and then incubated in an oven at 110 °C for 24 h. Once complete, the amino acid hydrolysate was dried in a rota-evaporator and then, the resulting sample was dissolved in 25 mL borate buffer (1 M, pH 9.0). Five millilitres of this sample were derivatised with 4 µL diethyl ethoxymethylene malonate at 50 °C for 50 min and shook vigorously. Of this sample, 20 µl was subsampled and injected directly into the HPLC system [174]. Of these three seaweeds tested, *M. pyrifera* had the second highest protein content at 13.2%. The amino acid profiles of *M. pyrifera* recorded the lowest content of amino acids of all three species at 0.8–1827.3 mg 100^−1^ g dry weight, with essential amino acids corresponding to 38.9% of total protein content [66]. 

#### 3.2.2. Phlorotannins and Polyphenol Extraction

To decipher the most effective phlorotannin extraction to perform on *M. pyrifera*, an orthogonal design set of experiments was performed previously [114]. Parameters important to optimal extraction conditions were investigated; these included pre-treatment use, solvent type, drying temperature, particle size, solid/liquid ratio, temperature, and extraction time. Recommended conditions included pre-treatment with hexane, extraction using water, 40 °C drying temperature, <1.4 mm particle size, extraction temperature of 55 °C for 4 h, and a solid–liquid ratio of 1:15 [114]. Using these parameters, phlorotannin content was 200.5 ± 5.6 mg gallic acid equivalent (GAE)/100 g for dry seaweed and total antioxidant activity was (TAA) 38.4 ± 2.9 mg Trolox equivalent TE/100 g for dry seaweed. Using HPLC-ESI-MS, two phlorotannins were detected: phloroeckol and tetrameric phloroglucinol [114]. More recently, *M. pyrifera* was investigated for its phlorotannin properties in another study. Extraction yields of carbohydrates and phlorotannins were 81.02 ± 8.9% and 1.62 ± 0.13% *w/w*, respectively. The phlorotannin fraction activity was concluded to be useful as a natural antioxidant and an antibacterial compound [175].

Four seaweed species, *Fucus serratus, L. digitata* (Ochrophyta, Phaeophyceae), *Gracilaria gracilis* (Rhodophyta), and *Codium fragile* (Chlorophyta), were tested for antioxidant activity and total phenol content (TPC) using solid–liquid extraction (SLE) and pressurised liquid extraction (PLE). These extraction methods were evaluated using 2,2-diphenyl-1-picrylhdrazyl (DPPH) and ferric reducing antioxidant power (FRAP) assays and the Folin–Ciocalteu total phenol content (TPC) assay. Results indicated that *Fucus serratus* had TPC and antioxidant activities thirty times higher than the other species. Only low TPC levels were observed for *L. digitata*, *G. gracilis*, and *C. fragile* from both SLE and PLE extracts, yet the SLE extracts did retain higher FRAP and DPPH activities than PLE extracts. This study concluded that pressures and high temperatures in PLE did not improve the antioxidant activities when compared to SLE extraction [176].

#### 3.2.3. Carbohydrate Extraction

Commercially viable fucoidan is extracted from a range of brown seaweed species including *M. pyrifera* and *Laminaria* sp. by a company in Tasmania, Marinova Pty Ltd. Marinova have developed a patented process called Maritech^TM^, which uses a coldwater extraction process that is species-specific, enabling up to 95% purity levels of fucoidan to be attained. The Marinova product is reported to retain optimal bioactivity due to the nature-like high molecular weight molecules, which can be used as components for cosmetic or functional food applications [145].

One method used to extract polysaccharides from *L. digitata* begins with a hot acid extraction applied to freeze-dried frozen samples. This process previously used powdered ground seaweed and suspended it in 0.1 M HCl at a ratio of 1:10 (*w/v*). The sample was incubated at 70 °C in an orbital shaker at 175 rpm for three successive time periods of 3, 3, and 24 h. Extracts were filtered using a muslin bag with the extract removed after each period and fresh solvent added to the retentate. All three extract batches were pooled and underwent further filtering with cotton and glass wool using a funnel, Buchner flask, and vacuum pump. The final filtrates were neutralised with the addition of 20 M NaOH and freeze-dried. An ethanol precipitation was undertaken to produce a crude protein extract (CE). This was compared with a depolymerised extract (DE), which had been put through a further purification using a Fenton reaction [98]. Dietary soluble and insoluble fractions were also measured. Results found both extracts produced microbiota-associated metabolic and compositional changes, indicating putative beneficial health benefits of *L. digitata* in vitro, yet further work is needed to clarify if fibre can positively alter the gut microbiota and cause health benefits in vivo [98].

Another study focused on extracting polysaccharides, laminarin, and fucoidan from *Saccharina japonica.* Their aim was to produce a simple quick and reliable method using high-performance size exclusion chromatography (HPSEC). The initial extraction was similar to the aforementioned *L. digitata* method that used a ground sample, which was re-suspended in 30.0 mL solvent in a flask. The suspension was heated and stirred constantly, then after cooling to room temperature, the suspension was centrifuged. An ethanol precipitation with a 3:1 ethanol to filtrate ratio was applied, sediments were washed twice using acetone then ether sequentially. The depurated sediments were dried at 60 °C and polysaccharides were obtained for further analysis. The polysaccharides were re-dissolved in the solvent. The extraction solution was then filtered (0.45 mm) prior to injection into the HPLC system. Each sample was injected, in triplicate, to assess the precision and accuracy of the analysis. Results produced 169.2 mg g^−1^ of fucoidan and 383.8 mg g^−1^ of laminarin [177]. Sodium alginate was the compound of interest when extracted from a Moroccan strain of *L. digitata*. Different conditions including temperature and sample size were used during the extractions. The alginates were purified by re-precipitation with ethanol and characterised by ^1^H-NMR, fluorescence spectroscopy, and infrared spectroscopy. The highest pure alginate yield 51.8% was reached using a < 1mm sample size and a temperature of 40 °C [83].

### 3.3. Pharmaceutical Extraction

#### 3.3.1. Protein Extraction

Proteins contain amino acids, essential amino acids, and peptides. Seaweed proteins account for between 5 and 20% [53]. Bioactive peptides activity has been linked to a range of health benefits including antimicrobial activity and blood pressure-lowering including angiotensin converting enzyme-1 (ACE-I) and renin inhibitory bioactivities, anti-atherosclerotic, antioxidant, antithrombotic, and immune-modulatory activities [178]. Ultrasound-assisted solvent extraction and using ultrasound as a pre-treatment before an acid or alkaline treatment have improved recovery of seaweed proteins [179]. Extracting bioactive peptides from these proteins requires hydrolysis to release functional peptide fragments with their specific bioactivities intact. Additionally, enzymatic hydrolysis is favoured by the pharmaceutical and functional food industry as it avoids severe chemical and physical treatments and retains both functional and nutritional properties [180,181]. To extract ACE and antioxidant peptides, proteolytic enzymes are used; flavourzyme, corolase PP, and other enzymes were reportedly used previously for hydrolysis, which resulted in the release of encrypted peptides which range in size from 2 to 20 amino acids. Further concentration and fractionation into their specific molecular weight cut off have achieved through a ultrafiltration membrane and gel permeation chromatography, yet ion exchange, affinity chromatography, and high performance liquid chromatography (HPLC) are the most vital chromatographic approaches that are employed [180,181,182].

Bioproteins and peptides have been extracted and characterised using the aforementioned procedures from the seaweeds *Ulva lactuca* (Chlorophyta), *Solieria chordalis, Palmaria palmata* (Rhodophyta), and *Saccharina longicruris* (Ochrophyta, Phaeophyceae) [183]. Enzyme-assisted extraction using cellulase optimised protein extract from *M. pyrifera*, which demonstrated antioxidant activity and potential antihypertensive activity [184]. To date, studies of bioactive activity from *L. digitata* have focussed on its carbohydrates, with no studies on protein or peptide bioactivity for pharmaceutical applications.

#### 3.3.2. Phlorotannins and Polyphenol Extraction

A study conducted on phlorotannins from *M. pyrifera* used macroporous resins as a purification method, with the potential application to expand the use of phlorotannins as a bioactive substance in the food, functional food, and pharmaceutical sectors. To prepare the *M. pyrifera* phlorotannins extract for purification, *M. pyrifera* was dried at 40 °C and ground to <0.5 mm. The dried sample was extracted using a solution 0.5 M of NaOH, solid/liquid ratio of 1/20 at 100 °C for 3 h. The mixer was filtered using Whatmann N^0^1 paper and the liquid phase was stored at 4 °C. The phlorotannin concentration present in the extract was 1800 mg of phloroglucinol equivalent (PGE)/L [185]. The six resins tested were Diaion HP-20, Sepabeads SP850, Amberlite XAD-7, XAD-16N, XAD-4, and XAD-2 (from Sigma-Aldrich). Prior to use, all resins were washed with 70% ethanol at 25 °C for 12 h. To test the adsorption of phlorotannins, a static adsorption was performed, using 2 g of each resin placed in a tube with 30 mL of phlorotannin extract. The tube was shaken in a shaking incubator, at 300 rpm, at 25 °C to reach adsorption equilibrium. After adsorption, the resins were filtered for the subsequent desorption of phlorotannins, and the concentration of phlorotannins in the extract was measured [186]. The phlorotannin concentration in the extracts was determined according to the Folin–Ciocalteu (FC) assay, and then adapted to a 96-well plate, with phloroglucinol concentrations ranging from 20 to 100 mg/L. The plate was then loaded with samples and standards (20 mL) separately, each well containing 100 mL of Folin–Ciocalteu’s reagent diluted with water (10 times) and 80 mL of sodium carbonate (7.5% *w/v*). The plate was then mixed and incubated at 45°C for 15 min. Absorbance was measured at 765 nm on a UV–visible spectrophotometer. The phlorotannin concentration was then determined by the regression equation of the calibration curve and expressed using mg of phloroglucinol equivalent (mg PGE/L). Phloroglucinol quantification was performed using a reverse phase C18 column (150 × 4.6 mm, 5 mm) and an HPLC system with an ultraviolet (UV) detector according to the method by [187]. Adsorption time for the resins on average was 9 h, the highest level of purification for phlorotannins was 42% with XAD-16N resin, with an adsorption capacity of 183 ± 18 mg PGE/g resin, and a desorption ratio of 38.2 ± 7.7%. The best temperature was 25 °C according to the adsorption isotherm; the Freundlich model best described the adsorption properties [186]. 

A study of antibacterial activity of phlorotannins from two brown seaweeds *Ascophyllum nodosum* and *Fucus serratus* used two methods to detect phlorotannin concentration, the aforementioned FC assay with detection using UV−vis spectroscopy, and second method ^1^H-NMR and ^13^C-NMR spectroscopy. The ^1^H-NMR and ^13^C-NMR spectroscopy method is both a qualitative and quantitative method which detects TPC and the linkages between phlorotannins present in the extract, which is then purified by solid phase extraction (SPE). Phenolic content was measured by quantitative NMR (qNMR) using milligram of phenolics per gram of seaweed (mg/g); however, the FC assay uses phloroglucinol equivalents per gram of seaweed (PGE/g). In this study, the concentration of phenolics in *Ascophyllum nodosum* was significantly higher than *Fucus serratus* at 37.35 and 17.00 mg/g, respectively, based on the ^1^H-NMR analysis. Conversely, the FC assay noted the opposite trend, with the phenolic content for *Ascophyllum nodosum* being 30.68 (±0.55) PGE/g, and *Fucus serratus* being higher in this analysis at 36.68 (±1.33) PGE/g. ^13^C-NMR spectra of the phenolic extract prepared using SPE from each species were compared to determine the difference in linkages of the phenolics between these seaweed species [188]. 

*L. digitata* has been investigated for its phlorotannin content [111]. The characterisation method used ^13^C and ^1^H-NMR spectroscopy for linkage characterisation and to determine extract purity. Phlorotannin fractions were obtained using NP-flash chromatography, followed by ESI-MS and MALDI-TOF-MS to decipher structural and molecular weight as well as identifying the fucol-to-phlorethol linkage ratio [111].

#### 3.3.3. Carbohydrate Extraction

*M. pyrifera*, although a member of the kelp family, *Laminariaceae*, it is typically not one of the kelp species investigated for pharmaceutical product content. Most studies in the literature focus on species from the *Laminaria* genus, such as *Saccharina japonica*, or *L. digitata*. A study analysed laminarian from *L. digitata* fucoidan, sodium alginate from *M. pyrifera*, and fucoidan from *Fucus vesiculosus* for biological activity, i.e., antitumour, cytotoxicity, and humoral immune response. Except fucoidan from *M. pyrifera*, all compounds were sourced already extracted from chemical companies, Kelco and Sigma. The fucoidan from *M. pyrifera* needed to be extracted. The extraction method for fucoidan used a dried milled sample from Argentina using a 30% ethanol extraction then split the sample into a dialysed and non-dialysed sample, then using the un-dialysed sample, split it into precipitate and supernatant. All extracts were tested in vivo in mice and in vitro for efficacy [189]. 

A patent filed in 2012 by the company Knocean Sciences, based in Texas, claimed it could provide total antioxidant protection using fucoidan extract from *M. pyrifera* at a commercial scale. Several products were proposed, and the extraction method used fresh kelp which was chopped and then milled before being pumped to a separation tank for 12–24 h, which enables gravity separation to liberate the kelp oil from the chopped milled kelp. The kelp oil is then drained and pumped to a holding tank, and the solid content at this stage is 4–7%. This kelp oil is then pumped to a thin film evaporator, then a wiped film evaporator, to pre-concentrate the solids in the kelp oil to 25–35% increasing viscosity. This was followed by a spray drying step, with an inlet temperature of 180 °C and an outlet temperature of 90 °C; this was optimised to the inlet/outlet feed temperatures, which can be increased to 225/105 °C with only a minimal loss of functionality and ORAC value (approximate 10% decrease) of the final product—a kelp concentrate powder. For the other products to be produced, the chopped and milled fresh kelp that is left over from the kelp oil separation process is then dried using a range of procedures including geothermal drying, wind drying, solar drying, and mechanical drying. This drying step continues until the product is 85–90% solids, usually 2–6 days, dependent on weather conditions. The dried product is placed in a hopper and fed into a milling system to produce a finely milled product which is tested for fucoidan content at this stage. This is the final product which is milled to the appropriate mesh size needed for the specific use; high purity fucoidan requires a mesh “size of 40”. The uses of this product range include as a food, dietary supplement, skin cosmetic, and pharmaceutical treatments for viruses or oxidation control [99]. Several *L. digitata* extract-based pharmaceutical products have been manufactured by two companies, Actipone^®^ and Boiron, yet extraction details were unavailable [100,101]. 

The goal of the exploration of seaweed polysaccharides with potential bioactive compounds is to prepare a crude seaweed extract for precise detection, identification, and quantification of target analytes applying chromatographic and spectrometric methods. This process includes efficient extraction, elimination of seaweed matrices, concentration, or dilution of the extracts. Quantitative chemical analysis of key groups of compounds in seaweeds are performed using chromatographic techniques, such as LC and GC coupled to MS and NMR, with TLC used for sample screening. When sample preparation and chromatography are appropriately selective, it is advantageous to rely on cheaper and more accurate spectroscopic detection procedures [190]. For successful seaweed product development, it is a necessity to become commercially viable; therefore, extraction methods must be both economical and environmentally friendly.

### 3.4. Extraction Improvements—Including Less Environmentally Impactful Strategies 

Protein has usually been extracted from seaweed using aqueous acid, or alkaline methods, or by enzymatic hydrolysis of dried powdered biomass. These proteins are then recovered through ultrafiltration and precipitation using ammonium sulphate or chromatography techniques [191]. To reduce the need for solvents’ enzymatic extractions, use enzymes such as proteases, cellulases, amylases, glucanases or endoproteases [192,193] to degrade the seaweed matrix structure and release the proteins. The presence of phenolic compounds, specifically phlorotannins in brown seaweeds, has a considerable influence on protein digestibility. Oven-drying and freeze-drying treatments were used on three brown seaweeds *Sargassum hemiphyllum, Sargassum henslowianum*, and *Sargassum patens*. Results found no significant differences in the amount of essential or individual amino acids from the two treatments; however, there was a significant difference in the total amino acids between treatments with the oven-dried samples significantly lower than the freeze-dried samples. In addition, properties such as swelling, water holding, and oil holding capacity of freeze-dried samples were significantly higher than the oven-dried samples, indicating the potential for freeze-dried seaweeds use as food ingredients in food products [194]. To improve the bioavailability of amino acids from seaweeds, heat treatments have been employed. One brown and red seaweed, *Alaria esculenta* and *Palmaria palmata*, were boiled for 15, 30, and 60 min. Results found an increase in accessible amino acids by 86–109% post treatment for the red seaweed *Palmaria palmata*; however, no equivalent results were noted for the brown seaweed *Alaria esculenta*, possibly due to its resilient cell wall physiology [195]. Other extraction methods also being investigated include chemical hydrolysis or subcritical water hydrolysis [192,196,197]. 

To reduce cost and extraction time, new methods such as ultrasound-assisted extraction (UAE), pulsed electric field (PEF), and microwave-assisted extraction (MAE) are being used. Ultrasound-assisted extraction (UAE) is a low cost and low solvent use method which is targeted towards high-value compounds, and works across a range of frequencies, from 10 to 20 MHz [198]; ultrasound creates bubbles which change in size depending on frequency, and creates increases in temperature of (5000 °C) and pressure of 2000 atmospheres. When the cavitation bubbles implode, the temperature and pressure released cause violent reactions which, if happening in proximity to cells, causes significant cell wall damage, if not complete cell rupture. If cavitation bubbles rupture in proximity to compounds, similar damage levels occur causing compound degradation and particle breakdown [198]. *Ascophyllum nodosum* protein extraction noted increased recovery when assisted by the use of ultrasound at an amplitude level of 68.4 μm as in a pre-treatment step; when compared with acid and alkaline treatment alone, recovery improved by 540% and 27%, respectively, and processing time was also reduced from 60 to 10 min [179]. Regarding commercial utilisation, UAE is used extensively in food processing at commercial scale, potentially enabling it to be used for seaweed protein extraction [199]. 

Pulsed electric field (PEF) is considered an emerging technology regarding intracellular extraction from seaweeds; it has been used effectively as a cell disruptor method for microalgae, yet its main use has been in lipid extraction to produce biofuels. Pulsed electric field (PEF) functions by the application of high electric currents to create holes in a cell wall or cell membrane, causing reversible or irreversible electroporation [200]. Initial studies had focussed on lipid extraction from green microalga, with one study which used the green seaweed *Ulva* for quantitative protein extraction, concluding that PEF was selective in its ability to extract and damage specific proteins [201,202]. It does show future potential for protein extraction from seaweeds due to the absence of both heat and solvents required by the method [203].

The use of MAE for protein extraction has been limited in seaweeds. The elevated temperatures up to 100 °C for several minutes coupled with high frequencies of 2450 MHz, creating bubbles under high pressure which can then rupture and disrupt the cell contents, may be effective at lipid extraction, but would not work for protein extraction [204,205].

Other methods which provide more promising solutions incorporate membrane filtration systems, which include the use of membrane technologies such as microfiltration, ultrafiltration, nanofiltration, and reverse osmosis. Filtration systems are controlled by the size of a particle, specifically their molecular weight, enabling exclusion of, for example larger to smaller particles, which differ in molecular weight. This system could be quite useful for the enrichment of algal proteins. These membrane filtration technologies do not create any thermal impact on the proteins and are environmentally friendly due to the lack of solvents used [206]. Additionally, regarding other potential applications, ultrafiltration has been utilised for polysaccharide purification for the brown seaweed *Sargassum pallidum* [207]. 

Polysaccharide extraction requires the liberation of these complex polysaccharides from the cell wall structure (Figure 1). The cell wall of seaweeds represents at a minimum 50% DW of the seaweed [62]. The cell wall structure is well known in seaweeds; these differences encompass numerous factors including differences in specific polysaccharide components based on the species, the physiological component of the algae being considered, the developmental and life cycle stage, as well as the season and habitat. Within seaweeds lineages, the classes of polysaccharides are extremely varied based on their degree of sulfation, esterification, molecular weight, and sugar residue configuration [168].

To date, the conventional extraction methods for polysaccharides used have included dilute aqueous acids, alkaline solutions, and other solvent-based extractions [144,208]. Newer methods include microwave, ultrasonic, hydrothermal, and enzyme-assisted methods, which have become established due to the increased yield, bioactivity, and the industrial and therapeutic applicability of seaweed polysaccharides. These technology-driven rather than chemical methods also assist in maintaining the chemical composition, their interior structure, and other vital properties. Aqueous-based extractions fulfil both the cost requirements and reduction in environmental impact for products using polysaccharides, but the efficiency of the yield is much lower than traditional chemical methods [209,210]. Other uses of aqueous-based extractions are present in the functional food sector, where conventional solvent extraction methods use chemicals such as chloroform, butanol, and hexane, which are not acceptable for these kinds of products. 

A study on brown seaweed *Ecklonia radiata* to improve antioxidant activities used microwave-assisted enzymatic extraction. The study found that in comparison to conventional acidic extraction, they had significantly higher yields in total phlorotannin content (TPC) and antioxidant activities, and an extraction yield of 52%. Utilising these two techniques in concert has created the opportunity for this brown seaweed to be used in producing value-adding nutritional products [211].

Microwave-assisted extraction (MAE) was used as an efficient and rapid method for the separation and purification of fucoxanthin from three seaweeds. The MAE method was used in conjunction with a high-speed counter current chromatography (HSCCC) system with a two-phase solvent system consisting of hexane-ethyl acetate-ethanol-water. Extraction from for *Saccharina japonica, Undaria pinnatifida*, and *Sargassum fusiforme* occurred in <75 mins for producing weights of 0.83, 1.09, and 0.20 mg of fucoxanthin, respectively. The fucoxanthin purity was 90% and detected using HPLC, with the structure further identified by liquid chromatography electrospray ionisation-mass spectrometry (LC-ESI-MS) and hydrogen 1-nuclear magnetic resonance (^1^H-NMR) [212]. Presently, the techniques being applied to extract polysaccharides from seaweeds are CSE, MAE, UAE, HAE, and EAE [209,213]. 

Liquid chromatography (LC) is used extensively in the detection of carbohydrates, yet for low quantities to separate carbohydrates and peptides, ion exchange chromatography (IEC) is used. To separate and then purify alginates and sulphated polysaccharides, anion-exchange chromatography (AEC) is used. Once purified, detection is performed using a range of methods including electrospray ionisation-tandem mass spectrometry (ESI-MS/MS) and matrix-assisted laser desorption/ionisation (MALDI) [214]. Ultra-performance liquid chromatography (UPLC) works similarly to high performance liquid chromatography (HPLC) but analyses particles of >2 μm in diameter to acquire better resolution, speed, and sensitivity than HPLC. It also reduces time and solvent use, making it a more attractive greener method when compared to HPLC [215]. Recently, UPLC was used to purify bioactive compounds and characterise amino acids from 21 seaweed species in Norway; it was also used to determine the monosaccharide composition of six seaweeds including *L. digitata* for their anticoagulant properties [216,217]. Using multiple chromatography methods is typical; for example, in the characterisation of fucoidan carbohydrates with anticancer activity from the brown seaweed *Padina boryana*, ion exchange chromatography (IEC) was followed by ESI-MS/MS [218]. To characterise carbohydrates, specifically to identify the number of monosaccharide units present, the resonances of ^1^H-NMR in the anomeric region (4.4–5.5 ppm) and the ^13^C-NMR spectra (95–110 ppm) provide crucial information [219].

Refinement and improvement in purification and detection methods have enabled more novel chromatographic and spectroscopy techniques to be used for characterisation and have been refined to tackle the complex structures of polysaccharides and protein from seaweeds. For future progression of seaweed product research and development, the utilisation of these novel techniques including UPLC, NMR spectroscopy (^1^H and ^13^C-NMR), ESI, and MALDI will be crucial [214]. 

#### Biorefinery Application—Optimised Biomass Utilisation

The manufacturing units of bio-economies are biorefineries. A biorefinery works with one or several feedstocks by providing biomass from which a range of products are produced. These products may include foods, food ingredients, agrochemicals, biomaterials, and biofuels [9]. When applying biorefineries to the marine environment, seaweed would be used as the main feedstock, and could provide alternative sources of biofuels, creating more sustainable alternatives to fossil-based resources [220]. Both *M. pyrifera* and *Laminaria* spp. have been previously investigated for their sustainable fuel-making capabilities, using anaerobic fermentation to produce methane or natural gas; additionally, *L. hyperborea* has also been studied for ethanol production [25,117,118].

As outlined previously, both *M. pyrifera* and *L. digitata* contain commercially viable polysaccharides, the alginates, which are used globally in the food, animal feed, and pharmaceutical industries [25], providing viable commercial products worth extracting as part of a biorefinery model. *Macrocystis* sp. and *Laminaria* sp. have been used to remove copper, zinc, and cadmium ions from solutions [119,120], potentially including these species as useful species in bioremediation. Based on the range of present commercial products produced from both, these brown seaweeds have potential to produce a biorefinery system for these species. For a future biorefinery for these brown seaweeds, the most crucial considerations to address would be that the biorefinery system being used to extract all products functions without significant loss in biomass, and a reduction in chemical solvent use.

Using *L. digitata* as a food and energy source within a biorefinery was investigated [221]. The study used enzymatic hydrolysis to solubilise the carbohydrates and convert them to simple sugars providing a 78.23% recovery of sugar in the seaweed hydrolysate. To aid production of a carbon source in the form of succinic acid, this seaweed hydrolysate was fermented using *Actinobacillus succinogenes* producing a yield conversion 86.49% (g/g^−1^ of total sugars) and an overall productivity of 0.50 g L^−1^ h^−1^; these results provide potential uses of *L. digitata* as a bio-based product and energy producer in biorefineries [221].

A recent biorefinery model study on the green seaweed *Ulva lactuca* used a cascading biorefinery process focussed on the protein extraction process and included extraction of five chemical products in this order starting with minerals, lipids, ulvan, protein, and cellulose [222]. The study found protein digestibility was 85% using an in vitro digestibility assay reaction of o-phthalaldehyde (OPA) and β-mercaptoethanol with primary amines, thus enabling the basis of a bioeconomy to be formed [222,223]. Another study on the green seaweed *Ulva lactuca* (formerly *Ulva fasciata*) (Chlorophyta) compared the direct and sequential extractions for mineral-rich liquid extract (MRLE), lipid, ulvan, and cellulose. They found that their sequential extraction system used 66% of the available biomass, while the direct extraction approach was less efficient, using between 3 and 30% of the biomass available [224].

An *M. pyrifera* study investigated the use of enhanced hydrolysis on *M. pyrifera* by integrated hydroxyl radicals and hot water pre-treatment (IHRHW) to produce monosaccharides or polysaccharides for use as potential fuel feedstocks. This pre-treatment method IHRHW uses a central composite design and is reported to be able to disrupt both cellulose and hemicellulose enzymatic hydrolysis barriers, preserve the pentose fractions, and minimize chemical demand and costs. The predicted optimum pre-treatment conditions were 113.95 °C for 29.1 min with the addition of 12.25 mM of ferrous sulphate. Enzymatic hydrolysis was performed on both pre-treated and untreated *M. pyrifera* using the desired 15FPU cellulase/g biomass loading. Hydrolysis was conducted in 50 mL Erlenmeyer flasks, with a 20 mL working volume. A 5% substrate concentration (*w/v*) was maintained. Sodium azide (0.5%) was added to the reaction mixtures to prevent microbial contamination. Samples were removed at regular intervals and the supernatants were boiled to denature enzymatic activity. Samples were then filtered using a 0.22 µm filter for glucose content analysis. Samples were incubated in triplicate at 50 °C and rotated at 150 rpm. A comparison was undertaken with untreated samples. Residues were separated from the liquid by centrifugation, decantation, and filtration after hydrolysis. The sugar in the liquid was then analysed. This enabled all glucan and xylan to be recovered as monosaccharides or polysaccharides and the overall yield was three times higher than the untreated samples of *M. pyrifera.* In addition, the digestibility achieved was 92.1% [225]. A recent investigation has claimed that phlorotannins, carbohydrate, and fertiliser fractions can be provided by using the biorefinery process on *M. pyrifera* [175]. This emerging method effectively works on disrupting the cellulose structure in the seaweed cell wall using hydroxyl radicals and without producing fermentation inhibitors, such as hydroxymethyl furfural or furfural. This high yield product is then ready for saccharification without requiring neutralisation. IHRHW provides an alternative to acid or alkaline pre-treatment, by reducing the utility cost, improving the yield, and being environmentally friendly. The attributes of this method make it a very attractive candidate for inclusion in a potential commercially viable seaweed biorefinery [225]. 

## 4. Regulation and Legislation

To ensure public safety when purchasing products, regulatory bodies such as the European Food Safety Authority (EFSA) in Europe and the US (FDA) publish lists of products or ingredients that are safe for consumption as food or functional food or pharmaceuticals and for use as novel foods or health ingredients.

In accordance with the online European Novel Food Catalogue, a list of twelve seaweed species (scientific and common names) are categorised as accepted for use as food (non-novel) and are not subject to Novel Food Regulation (EC) No. 258/97, or the updated Regulation (EU) 2015/2283; Novel Food Catalogue, 2018. This occurs due to specific seaweed species having a history of significant consumption as a food or food ingredient before 15 May 1997 in the EU. *L. digitata* is listed in this food catalogue as both a food and a non-food as of the 15 May 1997. However, *M. pyrifera* is not present in this European Novel Food Catalogue. To have a species added to this food catalogue list, the individual or company must undergo the authorisation process through Regulation 2015/2283, and normally, national authorities may assist with this process [5]. 

The FDA uses the designation GRAS (generally recognised as safe) to classify a substance to be used in food for humans or animals under Section 201 [226]. *M. pyrifera* and *L. digitata* have GRAS recognition for human consumption as flavour enhancers and flavour adjuvants [127], enabling their addition to functional foods being sold in the US.

Food regulations in France, have authorised 21 seaweed species including *L. digitata* and 3 species of the microalgae genus *Arthrospira*, formerly known as *Spirulina*, to be classified as vegetables and condiments. In relation to metal intake, defined maximum allowed values are as follows: lead (5 mg/kg), cadmium (0.5 mg/kg DW), tin (5 mg/kg DW), mercury (0.1 mg/kg DW), inorganic arsenic (5 mg/kg DW), and iodine (2000 mg/kg DW) [227]. An arsenic range (4–106 mg/kg) was found when ten seaweeds were surveyed at two locations in New England, USA. *L. digitata* was one of the ten seaweeds sampled and when extracted using a weak acid extraction with microwave heating, was found to contain high levels (2.8–20 mg/kg) of inorganic arsenic [228]. Another study of *L. digitata* and *Laminaria hyperborea* sampled on North Ronaldsay, one of the Orkney Islands off Scotland, detected arsenic of 74 ± 4 mg/kg DW, which formed the main diet for a sheep population on the island [229]. In the US, *L. digitata* is used in agriculture and livestock feed; therefore, monitoring of inorganic arsenic is recommended [228].

Fucoidan has been classified by the European Food Safety Authority (EFSA) as a novel food, making it a potential contender as a developing functional food ingredient [230]. The present extensive use of seaweed-derived fibres in the food industry confirms they are safe for human consumption, according to the European Food Safety Authority (EFSA) and the US Food and Drug Administration (FDA) [231,232,233]. Conversely, other seaweed fibres, including xylan, laminarin, and ulvan, have not received official EFSA approval to date [148]; consequently, their inclusion in food or functional food will be delayed. 

To safeguard human health, regulators have put guidelines in place for specific products. Prebiotic is not yet a term recognised by the US Food and Drug Administration (FDA). In the US, prebiotics are regulated based on the category of product their intent and design dictates [234]. 

In humans, to elicit an effect from the use of most prebiotics, an oral dose of >3 g per day is required [235]. Any dose lower than this should not be termed a prebiotic unless the low dose has been proven to produce specific effects on the microbiota and related health aspects [234]. 

## 5. Delivery Methods and Applications of Seaweeds 

### 5.1. Food and Feed Delivery and Applications

*L. digitata* and *M. pyrifera* have been approved as food flavour additives by the FDA as dehydrated or ground products, for direct addition to food for human consumption as a source of iodine or dietary supplement [127]. Delivery of these two seaweeds in food is in an unrefined form as dried seaweed and is added to food ingredients such as breadsticks and huiro fritters [63]. Delivery of seaweed extracts such as the phycocolloid, alginate, in food has been in the form of powder or gel, to be applied to biscuits, yoghurts, and ice creams, and as a food thickening and emulsifying agent [25,61,62]. The extracted polysaccharides of laminarin and fucoidan from *L. digitata* were delivered as wet and dry spray and applied to mince pork patties to improve appearance and reduce lipid oxidation [77].

The use of *L. digitata*, *L. hyperborea*, and *M. pyrifera* for animal feed was delivered as fresh seaweed and applied as the main food source for abalone and sea urchin and North Ronaldsay sheep; unrefined dried seaweed or flour seaweed was added as a dietary supplement for goats, bulls, rabbit, fish, and shrimp diets [14,19,20,21,22,23,67,68,69]. Extracted phycocolloid, called crude alginate, from *M. pyrifera* and *Laminaria* spp., was used in fish feeds as a binding agent [25]. 

Dietary supplements for sows and piglets were delivered using a seaweed extract from *Laminaria* sp. containing polysaccharides laminarin and fucoidan [70,73]. *L. digitata* also provided feed supplements for pigs through seaweed extracts containing laminarin and fucoidan, and purified β-glucans were added to their basal diets [74,75,76]. These polysaccharides were used as a prebiotic dietary supplement to improve the microbial gut populations of pigs, and specifically, weaning piglets [74,75,76]. 

### 5.2. Pharmaceutical Delivery and Applications 

*Macrocystis pyrifera* is used to treat thyroid conditions, anaemia in pregnancy in the US, and hypertension in Japan; an oral dose of 300 mg of iodine is recommended for hypertension treatment, with *M. pyrifera* reported to contain iodine at 0.1 to 0.5%. Medicinal preparations of iodine from *M. pyrifera* should be taken from the thallus [236]. Conversely, over consumption of seaweed was found to coincide frequently with medical ailments including goitre, hypothyroidism, and Hashimoto’s thyroiditis in countries where seaweed is used traditionally as food [237].

Alginates sourced from both *L. digitata* and *M. pyrifera* have been delivered in gels, powders, beads, and fibres and applied in wound dressings, indigestion control, and drug delivery [25,61,81,82,83]. With a growing global population, these alginate uses will continue to increase in demand and will undoubtedly require improved technology in their mode of delivery of these products. For example, the drug delivery speed or mode of action may need to be increased or consumed by a different method; in the case of wound dressings, alginates and sodium alginate are used in hydrogel which has the potential to contain bioactive compounds to improve the healing process [238]. 

Fucoidan has been extracted from *M. pyrifera, L. digitata*, and *Saccharina japonica*, and has shown bioactive properties, potentially making these fucoidans suitable to act as therapeutic agents for cancer and infectious diseases by assisting the immune system’s response, as an antitumour remedy, anti-inflammatory, antioxidant, and antibacterial, and to assist in lipid inhibition [89,239]. Fucoidan is a highly polar polysaccharide which limits its transport through the intestinal epithelial cells. Administration orally is then considered the easiest method; however, due to its molecular weight, oral bioavailability is considered low. To harness the clinical potential of fucoidan, a better understanding of preparation, quality standards, and administration must be acquired [60].

Secondary metabolites, phlorotannins extracted from several laminaria species including *S. japonica* and *L. hyperborea*, have been used in the effective control of human tumour cell proliferation, wound sealing, and reconstruction [112,113]. *M. pyrifera*, has also had two phlorotannins, phloroeckol and tetrameric phloroglucinol, identified as demonstrating antidiabetic and antioxidant activity as well as preventing skin aging [114]. Regarding application of phlorotannins, a study on the seaweed *Ishige okamurae*, which contains phlorotannins, suggested inclusion in potential functional food ingredients or nutraceuticals [138]. 

Seaweed extracts from *L. digitata* have been applied as a treatment to improve gut microbiota, and in commercial products as a skin moisturiser and as a homeopathic medicine [98,100,101]. A combination seaweed extract using *M. pyrifera, Fucus vesiculosus, Saccharina japonica*, zinc, and vitamin B6 was used to treat osteoarthritis, with positive results [115,116]. *M. pyrifera* seaweed extract in combination with krill oil was proposed to be useful as a pharmaceutical product with total antioxidant protection [99].

### 5.3. Other Applications

#### 5.3.1. Fuel

Three seaweed species were listed as potential fuel producers in Table 3: *M. pyrifera, Saccharina latissima*, and *Laminaria hyperborea* [25,117,118]. *Saccharina japonica*, another commercially grown brown seaweed species, has been investigated for its fuel potential using a novel engineered microbial platform. *S. japonica* was used as a model brown seaweed species, due to the high alginate content found in brown seaweeds and was fermented to produce ethanol. The novelty in this platform was the use of genetically modified *Escherichia coli* to produce an alginate-degrading enzyme called “Aly”, followed by consolidated bioprocessing (CBP), which incorporates enzyme production, with feedstock degradation (in this case, alginate) and metabolism, (through fermentation at a temperature range of 25–30 °C), resulting in an ethanol yield of ~4.7% *v/v* [240]. *S. japonica* has also been used as a feedstock for the production of bio-oil, gas, and char using fast pyrolysis, with the highest yield of 40.91 wt% at a temperature of 350 °C and sweeping-gas velocity of 300 mL/min [241]. Whether either of these processes will be implemented as a commercial fuel production system remains to be seen. Interestingly, *M. pyrifera*, like *S. japonica*, has been trialled for its fuel producing capacity using a similar process to CBP. A pilot study used 75 L fermentation of genetically modified *E. coli* on *M. pyrifera* biomass. The higher alginate to mannitol content in *M. pyrifera* required a four-stage process to exploit more of the carbohydrates present; this included acid leaching, de-polymerisation, saccharification, and fermentation steps, which yielded 0.213 kg ethanol kg^−1^ dry macroalgae, reaching 64% of the maximum theoretical ethanol yield [242]. Whether these systems will gain commercial success remains to be seen. More recently, the biorefinery process has been proposed and trialled in an attempt to utilise seaweeds, completely reducing any waste products during extraction [9]. This extraction system, which is still in the emerging technology stage, may enable a significant increase in seaweed biomass usage during extraction than a single product production allows [224]. The caveat is that the seaweed species being used in the biorefinery must produce a fuel feedstock. As *M. pyrifera* has already proven its potential as a fuel, this opens the utility of it as a biorefinery species.

#### 5.3.2. Nutricosmetics and Cosmeceuticals

Fucoidan’s well documented anti-inflammatory properties have been investigated as potential additional ingredients in nutricosmetics, with a focus towards anti-aging or sunscreen products. Marinova, an Australian-based seaweed bioproduct-producing company, utilises a cold water-based extraction for the polysaccharide fucoidan, which is supplied to the functional food and cosmetics industry [145]. Marinova also uses clinical trials to investigate the utility of fucoidan as anti-inflammatory and anti-aging ingredients in nutricosmetic products [243]. For potential nutricosmetics products, seaweeds and their extracts are well placed to be utilised for their inhibition of glycation, elastin calcification, and matric enzymes, as well as anti-inflammatory activity, all properties which assist in providing cosmetic anti-aging benefits [243]. 

Alginates extracted from *Macrocystis* sp. and *Laminaria* sp., and laminarins extracted from *Laminaria* sp., have been used in the cosmetics industry for their range of uses as gelling colloids and emulsion stabilisers for inclusion in lotions and moisturisers as well as immunostimulating, antioxidant, anticellulite, and anti-inflammatory agents [82,104,122]. There is further potential product development in this sector, with new applications of alginates and laminarins being explored and polysaccharides-rich extracts being included in skin care products to reduce the effects of aging and blemishes [244]. 

*L. digitata* (sea kelp) extract with glycerine added has been used to create a certified organic product by the USDA, which can be used in a range of products: anti-aging creams, serums, and face masks, hair treatments, root treatment, and shampoos [245]. Juice extracted from the whole *M. pyrifera* plant has been classified as a cosmetic ingredient for skin conditioning, now sold in several products including those for skin aging [246]. 

Interestingly, both *M. pyrifera* and *L. digitata* are listed in the FDA Product Category—Brown Algae-Derived Ingredients. *M. pyrifera* is listed in the ingredients of bath products, oils, salts, skin and eye products including masks, make-up, and self-tanning lotions, shampoo, foot products, nail lotions, nail polish, and aftershave, and its content ranges from 0.009 to 5% and has a higher content for eye lotions with 36.4%. *L. digitata* is also listed in the same products as *M. pyrifera* but has some additional products including hair bleaches, hair sprays, and for face and neck products, a concentration of 40%. Other product concentrations range from 0.0004 to 5% [127].

#### 5.3.3. Bioplastics

Sourcing bioplastics from *L. digitata* and *M. pyrifera* has not been documented to date; however, emerging uses for glycans extracted from the green seaweed *Ulva* include bioplastic. The Australian company Phycohealth already produces seaweed products including cosmetics, food, and food supplements and are behind this bioplastic product. The bioplastic they are working on is made using a glycan-based extract from *Ulva*, which is used to make thread which is then woven to produce a plastic film; other applications include potential 3D fabrication of materials [247]. 

#### 5.3.4. Bioremediation

Bioremediation of ions from metals is another use of both *M. pyrifera* and *L. digitata* [119,120,121]. In the context of aquaculture, the potential dual purpose of these species is to be cultivated as an aquaculture crop and be used to remove metals that may be a potential environmental hazard is promising. 

#### 5.3.5. Aquaculture

The addition of these species to an Integrated Multi-Trophic Aquaculture system (IMTA) could potentially improve the productivity of the seaweeds, while also producing multiple aquaculture products from one environmental footprint. The IMTA initial conceptual design is a system that allows several species to be grown within the same encloser system, utilising nutrients from each trophic level to the level below it. The system includes a fed aquaculture tank, for example, fin fish, beside a shellfish growing station (called organic extractive aquaculture). This takes advantage of the enrichment in particulate organic matter (POM) from the fish tank; next to the shellfish is the seaweed growing station, called the inorganic extractive aquaculture, which gains nutritional advantage from the dissolved inorganic nutrients (DIN) [248]. An integrated approach enables efficient nutrient cycling to take place, attempting to partially close the nutrient loop, reducing external nutrient supply to the system and improving efficiency, and minimising environmental impact to the local ecosystem by utilising the fish waste within the other trophic levels. Chopin described this system as extremely flexible, and it could be applied to land-based, freshwater, or marine, and could be termed “aquaponics”. Implementation of the IMTA system was trialled in Chile using the red seaweed *Agarophyton chilense* as a biofilter for nitrogen on an open-water salmon farm. The study found a significant reduction in nitrogen at 9.3 g M^−1^ per m of line. These long lines were positioned within 800 m of the salmon pens within the effluent flow. Seaweed tissue analysis for nitrogen noted up to 2% of the daily weight (DW) in the *Agarophyton chilense* that were growing within 800 m of the salmon pens. This site had the highest level of nitrogen in the seaweed tissue and the highest growth rates of up to 4% DW in summer and 2% DW in winter. This Chilean study concluded that IMTA was a successful biofiltration platform using red seaweed *Agarophyton chilense* and proposed that a 100 hectare (ha) *Agarophyton chilense* farm would effectively reduce the inorganic N inputs of a 1500-tonnes salmon farm [249].

## 6. Conclusions

Suggestions for the Future Potential of Commercial Brown Seaweed Cultivation

In the context of the future commercial viability of new seaweed products, it is important to factor in the commercially important phycocolloids sourced from seaweed which are alginates, agar, and carrageenan. The seaweeds from which these phycocolloids are extracted are *Ascophyllum, Durvillaea, Ecklonia, Lessonia, Laminaria, Macrocystis, Sargassum*, and *Turbinaria*. The brown seaweeds of interest in this review, *Laminaria* and *Macrocystis*, produce alginate. Alginate production is a global industry and was worth USD 213 million annually in 2009, yet none of the alginate-yielding seaweeds were produced by aquaculture at that time, as they were not grown by vegetative propagation. Cultivation of alginophytes, especially their reproductive cycles involving alternation of generation, requires more research. In China, *S. japonica* was the only alginate-producing seaweed to be cultivated primarily for food, while some excess was used for alginate extraction [250].

The seaweed industry requires large quantities and high-quality seaweed raw material that exerts pressure on the existing natural seaweed resources. Aquaculture cultivation of seaweed has grown considerably since 2009 to meet product demand and to protect wild kelp beds that are still under threat from over-exploitation and climate change, which has caused increases in ocean water temperatures [251]. To produce high value products (HVP) from seaweed requires considerable cultivation control to retain quality biomass, not possible in offshore systems; therefore, onshore aquacultures have been proposed to fulfil this need [252]. FAO reported that of the 29.5 million tonnes of seaweed harvested in 2016, only 0.5 million tonnes were wild-harvested, with 29 million tonnes from aquaculture [3]. Yet Chile, Peru, and Mexico still depend primarily on harvesting natural kelp beds [253]. Asia still dominates global seaweed production, extending productivity through cultivation of *Kappaphycus* and *Eucheuma* in Southeast Asia [3]. Further global development of seaweed aquaculture cultivation may benefit the preservation of wild kelp beds.

Additional suggestions have been made to further enable consistent successful aquaculture biomass production of seaweeds:

Extending knowledge of nutrient uptake and assimilation in the species of interest, which could be effectively employed when considering the utilisation of seaweed polyculture and Integrated Multi-Trophic Aquaculture (IMTA) [254]. Environmental conditions vary significantly in locations on the same coastline as has been demonstrated in Chile where biomass of *M. pyrifera* varied considerably based on local fluctuations in environmental variables and the occurrence of epiphytes [18]. Species-specific knowledge of nutrient uptake within the context of local environmental conditions could be vital in maintaining IMTA systems of cultivation and nutrient cycling within coastal systems.

To address concerns regarding the lack of nearshore space to accommodate future aquaculture sites as demand increases and due to the labour-intensive nature of seaweed harvesting, experts advise the use of offshore locations, where technology-based monitoring and harvesting can be carried out, reducing the necessity of a nearshore seaweed aquaculture location and labour-intensive harvesting. *Saccharina latissima*, a European kelp species, has been successfully cultivated offshore in Portugal in challenging conditions, producing growth rates of 3.3–4.5% day^−1^ from January to May [255]. To implement and maintain successful ongoing offshore seaweed cultivation systems, incorporation of technology is necessary.

Understanding of seaweed life cycles and where to control them is crucial to efficient aquaculture biomass productivity. Nori is the common name for the red seaweed *Porphyra purpurea* and nori production from *Pyropia*/*Porphyra* spp. is used as a successful example of this. The use of algal phytohormones to control the switch from vegetative-to-reproductive transition and to increase the speed of reproduction was performed on the red seaweed *Grateloupia imbricata* using methyl jasmonate. Maturation was reduced from three weeks to forty eight hours, and a 7.5-fold increase in cystocarp number was observed [256]. Further research into the understanding and manipulation of lifecycle stages and triggers to enhance reproductive success and speed of biomass accumulation should be considered.

A major challenge for the established commercially successful euchematoid (two genera of red seaweeds, *Kappaphycus* and *Eucheuma*, are known collectively as eucheumatoids) cultivation is the delay in the establishment of cultivars or strains with higher productivity and/or resistance to disease [257], which remains a challenge also for the brown and red seaweeds. Knowledge can be gained from progress made by other seaweed species. Using key commercial species, *S. japonica, Pyropia spp.*, *Undaria spp.*, *Cladosiphon okamurarus*, and *Nemacystus decipiens*, it took 21 years for Japan, Korea, and China to produce 47 certified seaweed cultivars to be commercially cultivated. These cultivars were produced to improve productivity; however, work is now continuing to produce higher quality seaweeds through aquaculture [258]. Enabling the expansion required for successful extensive bioproduct production from all three seaweeds groups will require the cultivation of domesticated seaweed cultivars, as well as the domestication of new species, and further streamlining of cultivation techniques, to include more comprehensive quality control methods, to enable higher quality seaweed bioproducts to be produced [6]. A temperature-tolerant mutant strain for *Neopyropia tenera* (formerly *Pyropia tenera*) was produced using gamma irradiation [259]. The potential to produce heat-tolerant seaweed strains for commercial seaweed species could become extremely important, with climate change affecting increases in ocean temperatures across the globe.

As an industry, the potential efficiencies that could be gained from these suggestions cannot be underestimated. The potential loss of natural kelp beds to over-exploitation and temperature increases due to climate change are serious concerns which need to be addressed in the immediate future. Independent cultivar cultivation and the development of species resistant to disease and epiphytes that are thermo-tolerant have the potential to significantly improve commercial opportunities for the seaweed industry.

## Figures and Tables

**Table 1:** Current commercial feed, food, and functional food products produced from *Macrocystis pyrifera* and *Laminaria* spp.

Brown Seaweed Species	Feed or Food	Food Group	Product Produced/Product Quality	Function of Product	Reference
*M. pyrifera*	Feed	Fresh seaweed	Fresh seaweed/ 9–13% Crude Protein	Food supply for abalone	[14,19,20]
*L. digitata*	Feed	Fresh seaweed	Fresh seaweed	Food supply for abalone and sea urchins	[14]
*L. digitata and L. hyperborea*	Feed	Fresh seaweed	1.4 ± 0.2 kg WW per day/79.6% Digestibility	North Ronaldsay sheep breed complete daily food supply; dry matter degradation (DMD, 71.7%, at 48 h)	[68]
*M. pyrifera*	Feed	Dried seaweed, included in meal	30% DW of Diet/77% Digestibility	30%DW of goat daily diet	[21]
*L. digitata*	Feed	Dried seaweed supplement to daily diet	Powder: 0.001 kg/day	Rabbits showed a significant effect of lowering total cholesterol, lipoprotein, especially triglyceride	[69]
*Saccharina japonica*	Food	Dried seaweed supplement	Powder (1, 2, 3, 4%)	Ash content increased; 1% seaweed added to breakfast sausages were the most improved for physiochemical and sensory properties	[64]
*M. pyrifera*	Food	Dried seaweed 3–28% Food recipe ingredient	Huiro fritters/6.9%DW of protein	Protein supplement	
*M. pyrifera*	Food	Dried seaweed 3–28% Food recipe ingredient	Breadsticks/9.5%DW of protein	Protein supplement	
					
*M. pyrifera*	Feed	*Macrocystis* meal food supplement	*Macrocystis* Meal concentration of (33.3 g/kg) based on shrimp weight, was fed (1.6 g over 28 days)/Protein efficiency ratio of 1.7 purified	Dietary supplement for juvenile white shrimp (*Litopenaeus vannamei*)	[67]
*M. pyrifera*	Feed	Complementary meal	Meal dietary supplement with 8.5% crude protein/85% Digestibility,	Dietary supplement for male bovine zebu bulls	[22]
*M. pyrifera*	Feed	Food supplement in the form of derived flour	3% DW of daily diet/low digestibility for salmonids fish	3% DW of daily diet as dietary supplement for minerals and carbohydrates needed by salmonids fish species	[23]
					
*M. pyrifera*	Food	Carbohydrates	Phycocolloids/ medium or high viscosity alginate	Thickening agent for food products	[25,61]
*M. pyrifera and Laminaria* spp.	Food	Carbohydrates	Phycocolloids/ Alginic acid powder	Ingredients for dietary biscuits, to induce feeling of fullness	[25]
*Laminaria* spp.	Food	Carbohydrates	Phycocolloids/ Alginate: soft to medium strength gel	Thickening agent for food products	[25,61]
*Macrocystis* sp.	Food	Carbohydrates	Phycocolloids/ Alginate	Emulsifying, gelling, stabiliser yoghurts, ice creams	[62]
*M. pyrifera and Laminaria* spp.	Feed	Carbohydrates	Phycocolloids/ crude alginate	Binding agent in salmon and other fish feeds	[25]
*Laminaria* spp.	Feed	Carbohydrates. Prebiotics: Seaweed extract as a dietary supplement	Polysaccharides: (Laminarin and Fucoidan) 1–4 g/kg/day	Supplement to daily diet weanling pigs for 21 days: alleviated the need to use of high-lactose diets for (>60 g/kg) for weanling pigs, and alleviated common problems occurring post-weaning	[70]
*Laminaria* spp.	Feed	Carbohydrates. Food Supplement	Polysaccharides: Laminarin (0.001 kg), Fucoidan (0.0008 kg), and ash (0.0082 kg) = Total supplement weight 0.01 kg/day	Enhanced piglet immune function and colonic microflora at weaning	[73]
*L. digitata*	Feed	Carbohydrates. Seaweed extract: dietary supplement: spray-dried (SD) and wet forms (WS)	Polysaccharides: Laminarin: 0.5 g/kg feed; Fucoidan: 0.42 g/kg Supplement to basal diet (Complete daily basal diet was SD = 1.9 kg/day; WS = 1.8 kg/day)	Reduction in lipid oxidation in the muscle tissue in 75% of pigs consequently improved the quality of pork steaks	[76]
*L. digitata*	Food	Carbohydrates. Seaweed extract: dietary supplement: spray-dried (SD) and wet forms (WS)	Laminarin (9.3%) and fucoidan (7.8%), added to mince pork patties	Reduced the appearance of surface redness of fresh patties, significantly decreased lipid oxidation in cooked patties	[77]
*L. digitata*	Feed	Carbohydrates. Dietary supplement seaweed extract	Polysaccharides: Laminarin and fucoidan: of 1.5 g/kg addition to the basal diet	Reduced the enterobacteria, bifidobacteria, and lactobacilli populations in the caecum and colon, while only marginal effects on the immune response was observed in weaned pigs	[74]
*L. digitata*	Feed	Carbohydrates. Dietary supplement	Structural polysaccharides: Purified β-glucans of 0.25 g/kg addition to the basal diet	Reduced the Enterobacteriaceae population and pro-inflammatory markers in the colon in pigs	[75]

**Table 2:** Current commercial pharmaceutical products from *Macrocystis pyrifera* and *Laminaria spp*.

Seaweed Types and Species	Compound of Interest	Product Produced	Function of Product	Reference
*M. pyrifera*	Phycocolloids/ medium or high viscosity alginate	Sodium and calcium alginate	Wound dressings	[25,81]
*M. pyrifera*	Phycocolloids/ Alginate	Alginic acid powder	Aid in relieving acid indigestion; treatment of gastroesophageal reflux (GERD) disease	[25,61]
*M. pyrifera*	Phycocolloids/ Alginate	Calcium alginate bead	Controlled release of medicinal drugs and other chemicals	[25,82]
*Laminaria* spp.	Phycocolloids/ Alginate: soft to medium strength gel	Sodium and calcium alginate fibres	Wound dressings	[25,81]
*Laminaria* spp.	Phycocolloids/ Alginate	Alginic acid powder	Aid in relieving acid indigestion; treatment of gastroesophageal reflux (GERD) disease	[25,61]
*Laminaria* spp.	Phycocolloids/ Alginate	Calcium alginate bead	Controlled release of medicinal drugs and other chemicals	[25,82]
*L. digitata*	Phycocolloids/ Alginate	Sodium alginate	Soft and elastic gels; potential drug delivery via microparticles	[83]
*M. pyrifera*	Sulphated polysaccharides	Fucoidan	Immune modulator, causing delays in apoptosis and promoting pro-inflammatory cytokine production	[89]
*Saccharina japonica*	Sulphated polysaccharides	Fucoidan	Significant reduction in thrombus lysis time	[88]
Brown seaweeds (including *L. digitata,* *Saccharina japonica*; *M. pyrifera*)	Sulphated polysaccharides	Fucoidan	Anti-inflammatory, antioxidant, antibacterial, and immunological activity; lipid inhibition; obesity prevention or treatment	[84,85,86,87]
*Saccharina japonica*	Secondary metabolite	Phlorotannin	Anti-proliferation of human tumour cells	[112]
*L. hyperborea*	Secondary metabolite	Phlorotannin	Wound sealing and reconstruction during wound healing	[113]
*M. pyrifera*	Secondary metabolite	Phlorotannin: phloroeckol, tetrameric phloroglucinol	Antidiabetic; antioxidant activity; prevention of skin aging	[114]
*L. digitata*	crude polysaccharide-rich seaweed extract	Crude extract and depolymerised extract	Improved gut microbiota composition; increase in short-chain fatty acids	[98]
*Fucus vesiculosus*, *M. pyrifera; Saccharina japonica*,	Seaweed extract: Maritech ^®®^ extract	*Fucus vesiculosus* 85%, *w/w*; *M. pyrifera* 10%, *w/w*; Saccharina *japonica*, 5%, *w/w*; zinc vitamin B6 and manganese)	Dose-dependent decrease in osteoarthritis in 5 females and 7 males	[115,116]
*M. pyrifera*	Seaweed extract	High purity Fucoidan 75–90% purity; Kelp Oil and/or Kelp Concentrate: Krill oil	Total antioxidant protection	[99]
*L. digitata*	Seaweed Extract: *L. digitata* thallus prepared in glycerine and water.	Actipone^®®^	A moisturising agent and stimulant, skin protecting, soothing, and smoothing properties.	[100]
*L. digitata*	Seaweed Extract: 1 DH	Boiron	Homeopathic medicine	[101]

**Table 3:** Other current uses of brown seaweeds *Macrocystis* sp. and *Laminaria* spp.

Seaweed Types and Species	Compound of interest	Product Produced	Function of Product	Reference
*M. pyrifera*,	Mannitol and alginate	Methane	Fuel	[25]
*Saccharina latissima*	Laminarian and mannitol, alginate	Methane: Natural Gas	Fuel	[118]
*L. hyperborea*	Laminarian and mannitol	Ethanol	Fuel	[117]
*Macrocystis* sp. and *Laminaria* sp.	Dried seaweed	Dried seaweed	Removal of copper, zinc, and cadmium ions from solution	[119]; [120]
*L. digitata*	Alginate	Beads covered in calcium alginate	Removal of heavy metals, cadmium, and copper from single and binary solutions	[121]
*Macrocystis* sp. and *Laminaria* sp.	Alginates	Alginates	Cosmetic uses: as gelling colloids, emulsion stabilisers, immunostimulating agents, moisturising, protective phycocolloids	[82,122]
*Laminaria* sp.	Laminarians	Laminarians	Cosmetic uses: antioxidant, anticellulite, and anti-inflammatory agents	[104]
*M. pyrifera*	Seaweed extract	High purity Fucoidan 75–90% purity	Antioxidant and health and wellness benefits (for potential use in cosmetics and nutraceuticals)	[99]
